# Comparative analysis of S100A10 and S100A11 in MASLD and hepatic cancer development revealed a tumor suppressive role for S100A10

**DOI:** 10.1038/s41419-025-07940-2

**Published:** 2025-08-21

**Authors:** Etienne Delangre, Marta Correia de Sousa, Miranda Türkal, Monika Gjorgjieva, Suzanne Chartier, Grégoire Arnoux, Cyril Sobolewski, Margot Fournier, Christine Maeder, Laura Rubbia-Brandt, Pierre Maechler, Michelangelo Foti

**Affiliations:** 1 Department of Cell Physiology and Metabolism, Faculty of Medicine, University of Geneva, Geneva, Switzerland; 2 Service de Pathologie Clinique, Hôpitaux Universitaires de Genève, Geneva, Switzerland

**Keywords:** Liver cancer, Non-alcoholic fatty liver disease, Tumour-suppressor proteins

## Abstract

S100 proteins are significantly deregulated in hepatocellular carcinoma (HCC) and metabolic dysfunction-associated steatotic liver disease (MASLD). Here, we investigated the impact of hepatocyte downregulation of two closely-related members of the S100 family, S100A10 and S100A11, in complementary mouse models of MASLD and liver cancer. Hepatotropic AAV8 encoding shRNAs targeting S100A10 or S100A11 were used to downregulate these proteins specifically in the liver of mice fed a diet inducing hepatic steatosis, inflammation, and fibrosis and in a genetic mouse model of MASLD bearing hepatocyte-specific deletion of PTEN (LPTENKO). The impact of S100A10 or S100A11 downregulation on liver tumor development was further investigated in aged LPTENKO mice spontaneously developing MASLD-driven HCC and in diethylnitrosamine (DEN)-injected mice fed or not with high fat diet. Finally, the upregulation and downregulations of S100A10 were performed in mice harbouring the over-expression of Myc and constitutively activated β-catenin, two main events occurring in a sub-type of human HCC. Downregulation of S100A10 promoted hepatocarcinogenesis in a fatty liver setting, while reducing steatosis and fibrosis development. S100A11 knock-down consistently reduced MASLD and tumoral growth. However, in vivo S100A11 downregulation triggered concomitant partial loss of endogenous protective S100A10. Overexpression of S100A10 reduced the volume of tumors and might represent a therapeutic option. The results show that both S100A10 and S100A11 play active roles in the development of MASLD. However, these two closely associated proteins present opposite contributions to hepatic cancer, S100A10 being protective and S100A11 deleterious.

## Introduction

Metabolic dysfunction-associated steatotic liver disease (MASLD) has emerged as a worldwide health burden driven by over-nutrition and sedentary lifestyle, contributing to obesity and type 2 diabetes incidence. MASLD, previously termed non-alcoholic fatty liver disease [1], originates from ectopic lipid accumulation in hepatocytes (simple steatosis), which can progress to more severe stages. Among them, metabolic dysfunction-associated steatohepatitis (MASH), a stage encompassing inflammation, hepatocyte ballooning, and collagen deposition (fibrosis), can prime the liver for the development of hepatocellular carcinoma (HCC) [2]. MASH and HCC are poorly curable and represent the 3rd cause of cancer-related deaths [3]. Indeed, surgical resection, transplantation, and chemotherapy remain sub-efficient, urging the need to identify novel therapeutic strategies. Concomitantly with the increased incidence of both MASLD and HCC, growing interest has emerged regarding the S100 calcium-binding protein family, particularly in inflammatory processes [4] and cancer [5]. Some of the S100 family members have already been reported to be increased in body fluids or in the liver of patients with HCC [6]. Among these S100 proteins, S100A10 and S100A11 appear as drastically upregulated in HCC and correlate with poor survival in patients [7, 8]. In the past years, S100A11 has been highlighted as one of the most promising targetable S100 proteins, with significant potential to mitigate liver disorders. Knock-down of S100A11 prevents steatosis as well as fibrogenesis [9] in short-term high-fat-diet (HFD)/methionine-choline deficient diet feeding or in CCl_4_-induced MASLD mouse models [8, 10, 11]. Nevertheless, the relevance of targeting S100A11 to treat MASH once the disease is already established or to hamper HCC development, has not been assessed so far. Although poorly investigated in the context of liver diseases, another member of the S100 family, S100A10, has been shown to be one of the main proteins present at the surface of lipid droplets in the liver [12] and to promote in vitro the growth of hepatoma cells lines [13].

In this context, we aimed to assess the therapeutic potential of targeting hepatocyte S100A10 and S100A11 in liver disorders. As no rodent model faithfully recapitulate the whole complexity of human MASLD and HCC, we investigated the roles of S100A10 and S100A11 using complementary mouse models of MASLD, MASLD-driven HCC, or liver cancer without metabolic disorders. The different mouse models with various etiologies could collectively recapitulate the human disease and shed new light on the roles of S100A10 and S100A11 in liver diseases.

## Material and methods

### Animal care and housing

Animals used in this study were male C57BL/6J mice or male liver-specific PTEN knockout mice (LPTENKO, AlbCre/PTEN^lox/lox^), as previously described [14]. Wild-type C57BL/6J were purchased from Charles River (France) for most protocols, except for the DEN and DEN-HFD protocols, which required in-house breeding (Zootechnie platform at the University of Geneva). An acclimation period of one month was implemented in the animal facility. Mice were kept in a conventional area, housed 2–5 per ventilated cages, under a controlled environment of 12 h/12 h dark/light cycle (7 a.m.–7 p.m.) and 20–24 °C. Mice received water and food *ad libitum* and the environment was enriched with a cardboard house, red plastic tunnel, nesting material, and wood sticks for teeth maintenance. For each protocol, mice were randomly allocated to different experimental groups. Experiments were conducted in accordance with the directives of the Swiss Federal Guidelines for Animal Experimentation, under authorization no. GE78, and ARRIVE guidelines. During the different procedures, animal weight and behavior were monitored. At the end of the different protocols, the animals were euthanized at 9 a.m. by isoflurane anesthesia, followed by decapitation. Blood was collected in heparin-coated tubes and plasma was isolated by centrifugation at 5000 rpm, 10 min, 4 °C. Organs were flash frozen in liquid nitrogen or processed for histological analysis. For each mouse, liver sample is composed of pieces from three lobes.

### AAV8 injection

Hepatotropic Adeno-Associated Viruses serotype 8 (AAV8) encoding shRNA control sequence (shCTL, U6 promotor), or shRNA directed against S100A10 mRNA (shS100A10, U6 promotor) or S100A11 mRNA (shS100A11, U6 promotor), or over-expressing mouse S100A10 (AAV8-CMV-m-S100A10, group OE-S100A10), were injected through retro-orbital injection under isoflurane anesthesia (2 × 10^11^ GC/mouse, 100 μL in NaCl 0.9%), in a conventional P2 area at the University of Geneva. After 10 days of recovery, mice were subjected to different procedures. For the long protocols, AAV8 were re-injected (half dose, 1 × 10^11^ GC/mouse, 100 μL in NaCl 0.9%) to ensure sustained knock-down (Fructose Palmitate Cholesterol diet 24 weeks, DEN, DEN-HFD, LPTENKO 6 months).

Because AAV8-based transduction triggers some degree of immunogenicity [15], eventually participating in the development of MASLD and HCC, all mice, including controls were injected with AAV8.

### Fructose, palmitate, cholesterol-enriched diet model

To induce MASLD/MASH, mice were fed a fructose/palmitate/cholesterol-enriched diet (FPC) supplemented with glucose and fructose in drinking water (42 g/l 55% glucose and 45% fructose). Mice were 10 weeks-old at the beginning of the feeding period, with an average body weight of approximatively 25–30 grams. The feeding period was 10 weeks to induce steatosis/inflammation or 24 weeks to promote a robust liver fibrosis. For the 24 weeks protocol, AAV8 were re-injected after 8 weeks of diet. Diets and drinking bottles were weighted and changed twice a week.

### Liver-specific PTEN knock-out (LPTENKO) model

*Pten* deletion in the hepatocytes was done using a Cre-recombinase expressed under the control of the albumin promotor as previously described [14]. Breeding was conducted in Charles River (France), while genotyping was assessed in-house. Shortly after weaning, mice were imported in the Zootechnie animal facility at the University of Geneva and kept in conventional housing conditions for adaptation.

#### Simple MASLD protocol

Four-months old LPTENKO mice were injected with AAV8 encoding shCTL, shS100A10, or shS100A11 and euthanized at 5 months-old to investigate short-term effect of S100A10/A11 knock-down on MASLD resolution.

#### MASLD-driven hepatocarcinogenesis protocol

6-months old LPTENKO mice were injected with AAV8 encoding shCTL, shS100A10 or shS100A11, re-injected at 9 months and sacrificed at 11 months. Mice were monitored for tumor number and size at 8, 9, 10, and 11 months-old by Magnetic Resonance Imaging (MRI) in vivo imaging. After sacrifice, tumors were counted in the explanted liver.

### DEN-induced HCC model

Fifteen days-old C57BL/6J were injected with Diethylnitrosamine (DEN) (25 mg/kg, 50 μL diluted in NaCl 0.9%). Half of the mice were fed with a standard diet, injected with AAV8 encoding shCTL, shS100A10, or shS100A11 at 7 months of age with a reinjection at 9 months of age, and sacrificed at 11 months-old. Tumor number and volume was monitored by Computed Tomography (CT)-scan in vivo imaging at 8, 9, 10, and 11 months old prior to sacrifice. The other half of DEN-injected mice was fed from 2 months old to 8 months old with a 60% HFD (E15742-34, Ssniff, Germany) to recapitulate hepatocarcinogenesis in a MASLD and obesity context. AAV8 were injected at 4 months-old and reinjected at 6-month-old. Tumor number and size was monitored by MRI in vivo imaging at 6, 7, and 8 months-old, prior to sacrifice.

### Transposase-based transfection of hepatocytes in vivo

Twelve-weeks old C57BL/6J mice were injected with AAV8 encoding shCTL, shS100A10, or for the over-expression of S100A10 (OE-S100A10). Hepatic carcinogenesis was induced through hydrodynamic injection (2 mL/5 s) of plasmids encoding for human Myc, human mutated β-catenin, leading to its constitutive activation and Sleeping Beauty transposase as previously described [16]. Tumor number and size was assessed at 3, 4, and 5 weeks post-hydrodynamic injection through CT-scan in vivo imaging.

### Blood parameters

Triglycerides were measured in plasma samples using the Cobas C111 system (ROCHE).

### Intra-hepatic triglyceride measurement

Liver samples (80–120 mg) were subjected to lipid extraction (Folch Method [17]). Lysis was performed in chloroform/methanol (2/1 v/v) using a Tissue-Lyser system (Qiagen). Colorimetric assay was performed to measure triglyceride concentration (Kit Triglycerides FS, DiaSys, ref 1 5710 99 10 021), reported on the weight of the tissue.

### CT-scan and MRI

Following retro-orbital injection of 100 µL ExiTron nano12000 (dilution ½, Viscover), mouse livers were imaged *via* computer tomography scan—Quantum GX microCT software (PerkinElmer). Analysis were performed using Imalytics software v3.1.1.6.

Following retro-orbital injection of 250 µL Primovist (dilution 1/10), mouse livers were imaged via MRI (Nanoscan 3 T, Mediso). Analysis were performed using Horos Software (v3.3.6, Horos Project).

### Proteomic analysis

Proteomic analysis was performed on the liver of LPTENKO-AAV8-shRNAs at 11 months of age. Protein samples were prepared using the phase-transfer surfactant method with minor modifications. Samples were reduced with 5 mM TCEP, alkylated with 20 mM iodoacetamide and digested with Lys-C and Trypsin at a 1:50 ratio. Digested peptides were desalted using MonoSpin C18 column and measured on an Easy nano LC—Orbitrap Fusion system (ThermoFisher Scientific, USA). Data were acquired in a data-independent acquisition mode and analyzed using the directDIA workflow in Spectronaut. FDR was estimated with the mProphet approach and set to 0.01 at both peptide precursor level and protein level. For two-group comparison, differential abundance testing was performed with unpaired *t*-test. *Q*-values were the multiple testing corrected *p*-values. The VolcaNoseR application was used to produce volcano plots using the proteomic data obtained. Deregulated proteins were identified using the following threshold: -log10(*q*-value) > 1, fold change to shCTL group = log2|0.5|. Over Representation Analysis by KEGG pathways was performed using shinyGO 0.81 application (accessed December 2024). The list of deregulated proteins identified in LPTENKO-shS100A10 and LPTENKO-shS100A11 groups was used in CancerMine database (accessed December 2024) to classify them as oncogenes/driver (ONC/D) or tumor suppressors (TS) based on at least 5 publications. Proteins with reported ONC/D and TS function were classified as “Dual”.

### Histology

Samples were collected from three different hepatic lobes and livers were fixed in 4% paraformaldehyde, embedded in paraffin, and cuts of 5 μm thickness were performed. Hematoxylin-Eosin and Masson’s Trichrome coloration were performed by the Histology Core Facility of the University of Geneva. When required for tumor classification, reticulin coloration was performed by the pathology department of the University Hospital of Geneva (HUG).

### Immunohistochemistry

Paraffin removal followed by rehydration (EtOH 100%, 100%, 95%, 70%, H_2_O) was applied on liver histological sections before antigen retrieval (citrate buffer, pH = 6, 30 min). Endogenous peroxidases were inhibited by 15 min incubation with H_2_O_2_ 3%. Blocking in 10% normal goat-serum followed by overnight incubation with anti-Iba1 (1/2000, Abcam, ab178846), anti-γH2AX (1/100, Cell Signaling, ref 9718), anti-S100A10 (1/100, Abcam, ab76472), or anti-S100A11 (1/100, Protein Tech, ref 10237-1-AP) primary antibodies. HRP-conjugated antibodies (1/200, BIORAD) allowed DAB-based staining (Abcam, ab64238). S100A10 and S100A11 staining was performed on human Tissue Micro-Array (LV1505a unstained slide, http://tissuearray.com).

### Semi-automatic quantification using QuPath

Whole slide images were analyzed with QuPath version 0.3.2 or 0.4.4 [18] using different semi-automatic tools available, manual curation was performed to validate and correct automated detections. In brief, liver sections were automatically identified using the “Simple tissue detection” feature. For brightfield images, each chromogenic stain combination was unmixed.

#### Fibrosis quantification

Masson’s trichrome-positive areas were delineated using pixel thresholding, and results were expressed as coverage of Masson-positive area over total area of the tissue.

#### Iba1 staining

DAB-positive areas were identified using pixel thresholding, and results were expressed as coverage of Iba1-positive area over total area of the tissue.

#### γH2AX staining

DAB-positive areas were identified using pixel thresholding, and results were expressed as percentage of γH2AX nuclei over total number of nuclei.

#### LD quantification

Lipid droplets were segmented using Cellpose [19]. Specifically, a custom-trained model was applied to the average of the red, green, and blue channels. The total area, the number, and the size of lipid droplets were computed. Finally, results were expressed as lipid droplets (coverage) area on total area of the tissue, lipid droplet number on total area of the tissue (density), and size of every single lipid droplet. Codes can be accessed upon request.

### Cell culture and transient over-expression of plasmids and siRNAs

#### Cell lines

Huh7 cells were purchased from Sekisui Genotech (JCRB0403, Japan) and cultured in DMEM (1 g/l glucose, Gibco, 21885-025) supplemented with 1% penicillin-streptomycin (Gibco, 15140122) and 10% fetal bovine serum (Gibco, A5256701). For the downregulation of S100A10 and S100A11, 200,000 cells were seeded in a 6-well plate and subjected to siRNA transfection during 72 h (20 nmol of siRNAs using the HiPerFect Transfection Reagent kit, Qiagen, ref 301704). Control siRNA (AllStars Neg.Control siRNA, Qiagen, ref. 1027310), anti-human S100a10 siRNA (Qiagen, ref. SI03246670), and anti-human S100a11 siRNA (Qiagen, ref. SI03132878) were used. For the over-expression of FLAG constructs, 200,000 cells were seeded and transfected during 48 h with empty pCAG vector, S100A10-FLAG, or S100A11-FLAG using Lipofectamine 3000 Transfection Reagent (ThermoFisher Scientific, L3000001). Cells lines were tested for mycoplasma contamination prior the start of the experiments.

#### Mouse primary hepatocytes (MPH)

Primary hepatocytes were isolated from 3 months old C57BL/6J mice as previously described [20]. 400,000 cells were seeded in a 6-well plate and subjected to siRNA transfection during 72 h (20 nmol of siRNA using the HiPerFect Transfection Reagent kit, Qiagen, ref 301704). siRNAs control (AllStars Neg.Control siRNA, Qiagen, ref. 1027310), mouse anti-S100a10 siRNAs (Qiagen, ref. SI01409233), and mouse anti-S100a11 siRNA (Qiagen, ref. SI01409268) were used.

### Co-immunoprecipitation

Huh7 cells were lysed in lysis buffer (150 mM NaCl, 50 mM Tris-HCl pH = 7.4, 1% Triton-X-100, 2 mM EDTA, 10 mM NaF, 2 mM NaVo4, 1 mM AEBSVF and protease inhibitors). Protein quantification was performed using cell lysates (BCA kit, ThermoFisher Scientific, ref A55864). For immunoprecipitation, proteins (200 μg) were loaded onto Protein G Sepharose previously saturated with 1% BSA 1% Triton-X-100 and anti-FLAG antibody (Sigma, ref. F3165) or mouse IgG (Sigma, I-5381).

### RNA extraction and RT-qPCR

RNA was extracted using the Trizole-chloroform-isopropanol method. 1 µg of RNA was used to synthesize cDNA (High-Capacity RNA-to-cDNA kit, Applied Biosystems, ref 4387406) prior to qPCR using PowerUp^TM^ SYBR^TM^ Green Master Mix (Applied Biosystems, ref A25742). qPCR were run in triplicate on a QuantStudio^TM^ 5 Real-Time PCR System (Applied Biosystems). The list of primer used is available in Table 1.

**Table 1:** List of primer used for qPCR.

Name	Organism	Forward Sequence (5′-3′)	Reverse sequence (5′-3′)
S100A10	Mouse	TGGAAACCATGATGCTTACGTT	GAAGCCCACTTTGCCATCTC
S100A11	Mouse	TGCCTACAGAGACTGAGAG	GGATCCTTCTGGTTCTTTGTG
Cyclophilin A	Mouse	CAAATGCTGGACCAAACACAA	GCCATCCAGCCATTCAGTCT
CD3	Mouse	ATG CGG TGG AAC ACT TTC TGG	GCA CGT CAA CTC TAC ACT GGT
CD8	Mouse	AAGAAAATGGACGCCGAACTT	AAGCCATATAGACAACGAAGGTG
CD4	Mouse	TCCTAGCTGTCACTCAAGGGA	TCAGAGAACTTCCAGGTGAAGA
TNFα	Mouse	GGCTGCCCCGACTACGT	GACTTTCTCCTGGTATGAGATAGCAA
IL-1β	Mouse	GACAACTGCACTACAGGC	CATGGAGAATATCACTTGTTGG
TGF-β	Mouse	GCCTGAGTGGCTGTCTTTTGA	GCTGAATCGAAAGCCCTGTATT

### Western blot

Proteins were extracted from livers, mouse primary hepatocytes, or Huh7 cells in RIPA buffer prior to protein quantification (BCA kit, ThermoFisher Scientific, ref A55864). 10 µg of proteins were charged on a 15% acrylamide gel, transferred to a nitrocellulose membrane (ThermoFisher Scientific, ref 77010), and saturated in 5% milk. S100A10 (1/2000, Abcam, ab76472), S100A11 (1/1000, Proteintech, ref 10237-1-AP), and tubulin (1/5000, Cell Signaling, ref 2128) antibodies were used prior HRP-conjugated secondary antibodies and chemiluminescence measurement (Fusion FX, Vilber).

### Data representation and statistical analysis

Data are represented as mean +/− S.E.M. For histological analyses, tumor number and size quantification, QuPath analysis, blood parameters, histopathological interpretation, researchers were blinded to each experimental group. “*n*” represents the number of mice, tumor, liver biopsies, or the number of independent experiments carried out in vitro. For the number of animals used in the design of the study, Power test was performed based on our previous experiences with an expected difference of 40% on steatosis, fibrosis, and tumor numbers (effect size 1.11; power: 0.80). Outliers test was performed using the ROUT method (*Q* = 1%). For batch analysis, intra-experiment loss of sample (hemolysis, destruction of the sample, loss of mice, or outliers) resulted in variability in the number of samples. In addition, in some of the protocols, animals were excluded at certain time points when the endpoints stated in the experimental authorization were reached. Normal distribution and variances were tested using the Shapiro–Wilk normality test and Fisher test. Statistical significances were calculated using One-Way ANOVA, Two-Way ANOVA, *t*-test, or non-parametric equivalent. Multiple comparison of Dunnett was used when more than 2 groups were compared using Graph-Pad Prism 9 software. *P*-values below 0.05 were considered significant.

## Results

### Downregulation of S100A10 and S100A11 reduces MASH development induced by a Fructose Palmitate Cholesterol-enriched diet

**Fig. 1:**
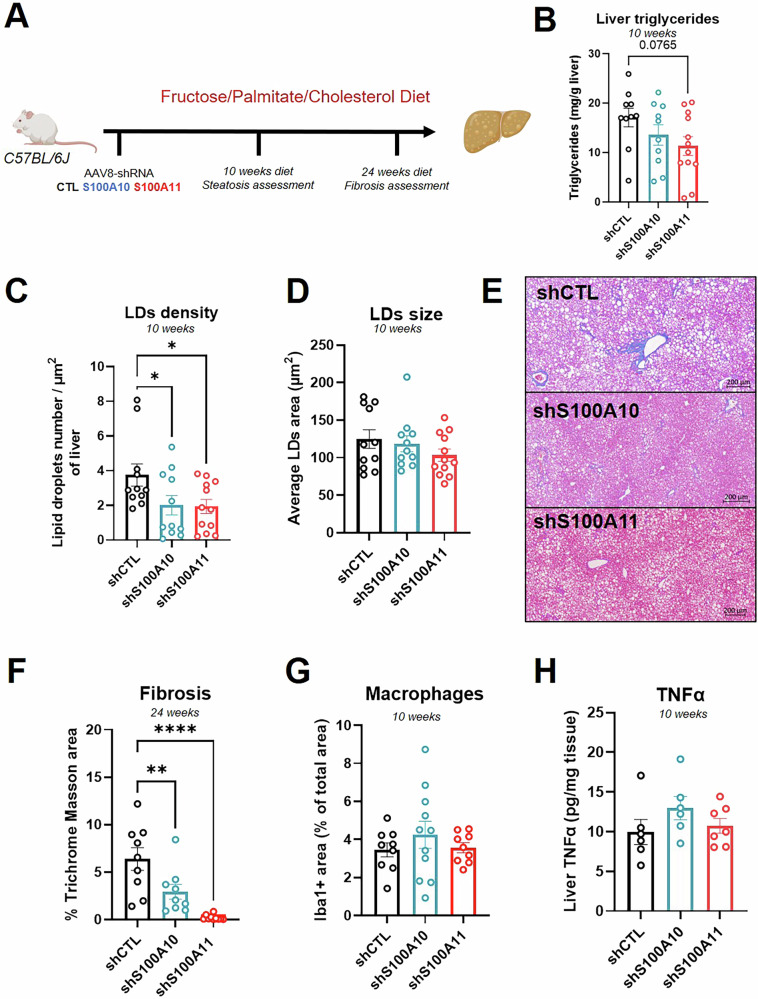
Downregulation of S100A10 and S100A11 reduces MASH development induced by a Fructose Palmitate Cholesterol-enriched diet. **A** Experimental design of the fructose/palmitate/cholesterol-enriched diet (FPC) protocol, including feeding period and AAV8 injections. **B** Liver triglyceride content measured by colorimetric method following Folch’s extraction of total lipids. C57BL/6J mice were injected with AAV8-shCTL (*n* = 10), AAV8-shS100A10 (*n* = 10), or AAV8-shS100A11 (*n* = 12) and fed during 10 weeks with FPC. **C** Automatic detection of lipid droplets numbers in the livers of C57BL/6J mice injected with AAV8-shCTL (*n* = 11), AAV8-shS100A10 (*n* = 11), or AAV8-shS100A11 (*n* = 12) and fed for 10 weeks with FPC. Results are normalized to total tissue area. **D** Automatic detection of individual lipid droplets size in the livers of C57BL/6J mice injected with AAV8-shCTL (*n* = 11), AAV8-shS100A10 (*n* = 11), or AAV8-shS100A11 (*n* = 12) and fed for 10 weeks with FPC. Results are expressed as the average lipid droplet size per mouse. **E** Representative images of Masson’s Trichrome coloration in the liver of C57BL/6J mice injected with AAV8-shCTL, AAV8-shS100A10, or AAV8-shS100A11 and fed for 24 weeks with FPC. Scale bar: 200 µm. **F** Automatic detection of collagen deposition extent based on Masson’s coloration in the liver of C57BL/6J mice injected with AAV8-shCTL (*n* = 9), AAV8-shS100A10 (*n* = 9), or AAV8-shS100A11 (*n* = 10) and fed for 24 weeks with FPC. Results are expressed as the percentage of Masson’s Trichrome positive area on the total area of the tissue. **G** Quantification of macrophages density measured by Iba1 immunohistochemistry in the liver of C57BL/6J mice injected with AAV8-shCTL (*n* = 9), AAV8-shS100A10 (*n* = 11), or AAV8-shS100A11 (*n* = 9) and fed during 10 weeks with FPC. Results are expressed as the percentage of Iba1+ area over the total tissue area. **H** Liver TNFα content measured by ELISA in C57BL/6J mice injected with AAV8-shCTL (*n* = 6), AAV8-shS100A10 (*n* = 6), or AAV8-shS100A11 (*n* = 7) and fed for 10 weeks with FPC. Results are presented as means +/− S.E.M. “*n*” represents the number of animals. * = *p* < 0.05; ** = *p* < 0.01; **** = *p* < 0.0001 determined by One-Way ANOVA followed by Dunnett’s post-hoc analysis.

To investigate the role of S100A10 and S100A11 in MASLD/MASH, C57BL/6J mice were fed with a Fructose Palmitate Cholesterol-enriched diet, mimicking human steatohepatitis [21]. S100A10 and S100A11 were downregulated in the hepatocytes using hepatotropic AAV8 encoding for control shRNA sequence (shCTL) or shRNAs targeting the mRNA of S100A10 (shS100A10) or S100A11 (shS100A11) (Fig. 1A). Knock-down extent was around 90% after 3 weeks of AAV8 injection (Fig. S1A and [8]) and about 50% at 10 weeks post-injection (Fig. S1B, C). The livers of mice that underwent 10 weeks feeding period were assessed for steatosis and inflammation, while 24 weeks of FPC diet was needed to induce a massive hepatic fibrosis (Fig. 1A). After 10 weeks of FPC diet, S100A10 or S100A11 knock-down reduced steatosis development, assessed by the measurement of liver triglyceride content (Fig. 1B) and by automatic quantification of lipid droplets density on H&E coloration (Fig. 1C). However, the size of individual lipid droplets was unaffected (Fig. 1D). Fibrosis development was strikingly reduced with the knock-down of S100A10 and barely detected in S100A11 downregulated mice after 24 weeks of FPC diet, highlighting a crucial role of S100A11 in MASH development (Fig. 1E, F). Inflammatory processes link steatosis/liver injuries to fibrosis. In this model, downregulation of S100A10 or S100A11 did not mitigate macrophage infiltration or hepatic TNFα (Figs. 1G, H and S1D, E), arguing against an inflammatory contribution of S100A10 or S100A11. After 24 weeks of diet, macrophage coverage was even increased in the shS100A11 group. This is supported by RT-qPCR analysis of key mediators of liver inflammation (Fig. S1F).

Overall, in a diet-induced MASLD/MASH model, targeting hepatic S100A11 emerges as a more effective therapeutic option compared to S100A10, although S100A10 downregulation also showed beneficial effects.

### Prolonged S100A10/A11 silencing shows a therapeutic benefit on steatosis in a genetic model of MASLD-driven liver cancer

**Fig. 2:**
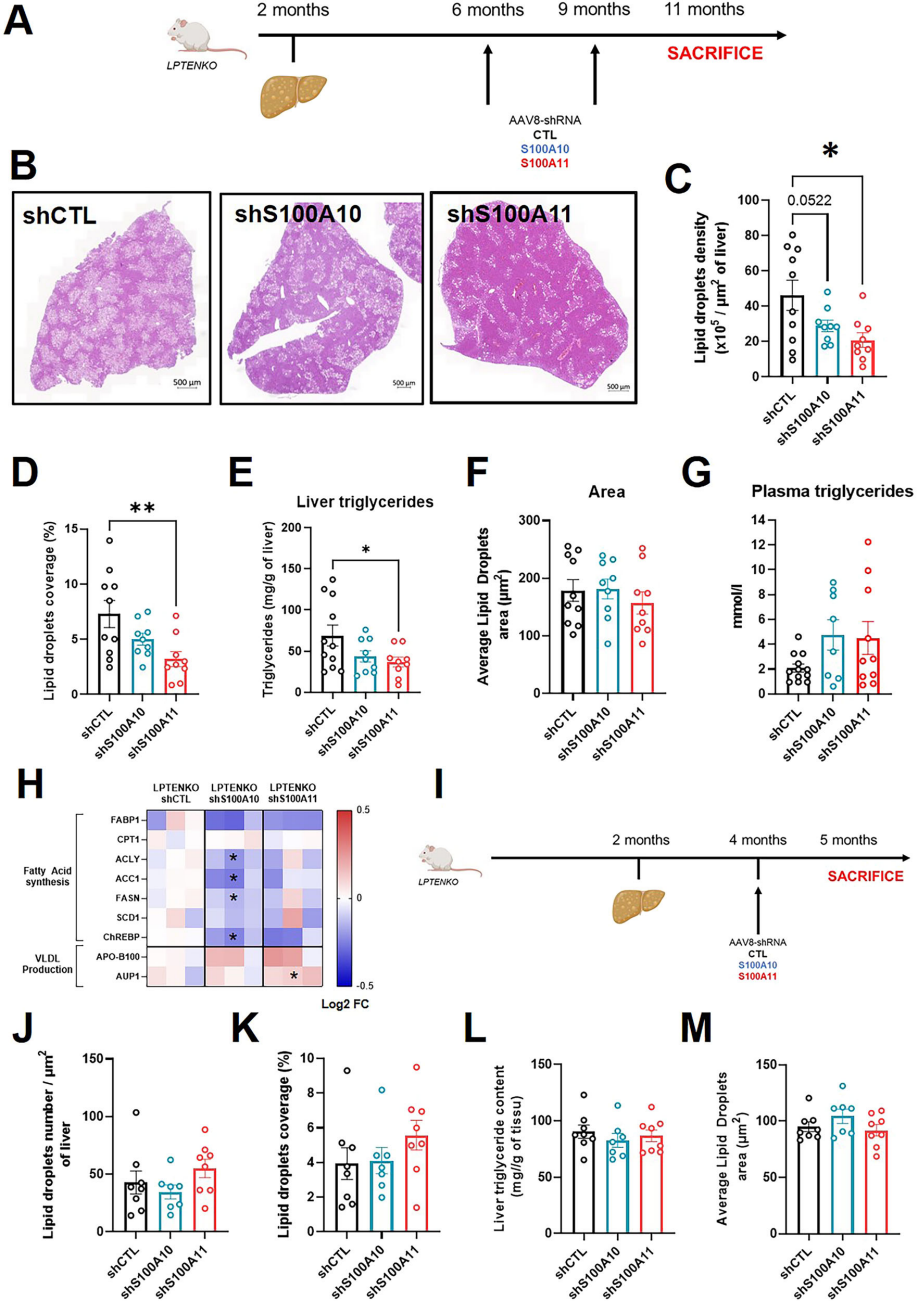
Prolonged S100A10/A11 silencing shows a therapeutic benefit on steatosis in a genetic model of MASLD-driven liver cancer. **A** Experimental design of the long-term LPTENKO protocol, including the spontaneous appearance of steatosis, AAV8 injections, and sacrifice. **B** Representative images of hematoxylin-eosin coloration in the livers of LPTENKO mice injected with AAV8-shCTL, AAV8-shS100A10, or AAV8-shS100A11 at 11 months of age. Scale bar: 500 µm. **C** Automatic detection of lipid droplets number in the liver of LPTENKO mice injected with AAV8-shCTL (*n* = 10), AAV8-shS100A10 (*n* = 9), or AAV8-shS100A11 (*n* = 9) at 11 months of age. Results are normalized to total tissue area. **D** Automatic detection of lipid droplets coverage in the liver of LPTENKO mice injected with AAV8-shCTL (*n* = 10), AAV8-shS100A10 (*n* = 9), or AAV8-shS100A11 (*n* = 9) at 11 months of age. Results are normalized to total tissue area. **E** Liver triglycerides content measured by colorimetric method following Folch’s extraction of total lipids. LPTENKO mice were injected with AAV8-shCTL (*n* = 11), AAV8-shS100A10 (*n* = 9), or AAV8-shS100A11 (*n* = 9) at 11 months old. **F** Automatic detection of lipid droplets size in the liver of LPTENKO mice injected with AAV8-shCTL (*n* = 10), AAV8-shS100A10 (*n* = 9), or AAV8-shS100A11 (*n* = 9) at 11 months of age. Results are expressed as the average of lipid droplets size per mouse. **G** Plasma triglycerides levels measured in LPTENKO mice injected with AAV8-shCTL (*n* = 12), AAV8-shS100A10 (*n* = 8), and AAV8-shS100A11 (*n* = 10) at 11 months-old. **H** Heatmap representation of proteomic analysis performed in the liver of LPTENKO injected with shCTL (*n* = 3), shS100A10 (*n* = 3), or shS100A11 (*n* = 3) for protein involved in fatty acid synthesis or VLDL production/export at 11 months of age. **I** Experimental design of the short-term LPTENKO protocol, including the spontaneous appearance of steatosis, AAV8 injection, and sacrifice. **J** Automatic detection of lipid droplet numbers in the liver of LPTENKO mice injected with AAV8-shCTL (*n* = 8), AAV8-shS100A10 (*n* = 7), or AAV8-shS100A11 (*n* = 8) at 5 months of age. Results are normalized to the total tissue area. **K** Automatic detection of lipid droplets coverage in the liver of LPTENKO mice injected with AAV8-shCTL (*n* = 8), AAV8-shS100A10 (*n* = 7), or AAV8-shS100A11 (*n* = 8) at 5 months of age. Results are normalized to total tissue area. **L** Liver triglycerides content measured by colorimetric method following Folch’s extraction of total lipids. LPTENKO mice were injected with AAV8-shCTL (*n* = 8), AAV8-shS100A10 (*n* = 7), or AAV8-shS100A11 (*n* = 8) at 5 months of age. **M** Automatic detection of lipid droplets size in the liver of LPTENKO injected with AAV8-shCTL (*n* = 8), AAV8-shS100A10 (*n* = 7), or AAV8-shS100A11 (*n* = 8), at 5 months of age. Results are expressed as the average lipid droplets size per mouse. Results are presented as means +/− S.E.M. “*n*” represents the number of animals. * = *p* < 0.05; ** = *p* < 0.01 determined by One-Way ANOVA followed by Dunnett’s post-hoc analysis (**C**–**G**, **J**–**M**) or by *t*-test (**H**).

To further investigate the therapeutic potential of S100A10 and S100A11 in MASLD and HCC, we used the LPTENKO genetic model, covering simple steatosis to HCC development. Steatosis develops around 2 months of age in LPTENKO as described previously [22], whereas tumorigenesis arises around 7 months (adenomas) and HCC at 14–15 months [14]. From 6 to 11 months of age, after steatosis development, LPTENKO mice were subjected to AAV8-based S100A10 or S100A11 downregulation (Fig. 2A). As a result, lipid droplet density (Fig. 2B, C) and coverage (Fig. 2D) were lowered by 30–40% in S100A10 and significantly decreased by 50–60% in S100A11-knocked down mice, correlating with the reduced intra-hepatic triglyceride content (Fig. 2E). The area of individual lipid droplets was unaffected (Fig. 2F). Intriguingly, LPTENKO mice with knock-down for S100A10 or S100A11 displayed a trend for elevated triglyceride levels in the plasma, suggesting an effect on very low-density lipoprotein (VLDL) homeostasis (Fig. 2G).

To deepen our understanding of the molecular networks affected by the knock-down of S100A10 or S100A11, we performed proteomic analysis in liver samples from LPTENKO groups (shCTL, shS100A10, and shS100A11). We identified 281 deregulated proteins in the shS100A10 group and 493 in the shS100A11 group (Fig. S2A). Gene enrichment analysis of deregulated proteins in the two groups pinpointed toward pathways involved in fatty acid synthesis and degradation (Fig. S2B–D). Indeed, knock-down of S100A10 was associated with decreased expression of fatty acid synthesis component (Fig. 2H). In addition, the proteomic analysis revealed that VLDL formation/export machinery was upregulated in shS100A11 mice (Fig. 2H). Although LPTENKO mice do not present fibrosis at this age, we measured collagen deposit in the liver and found no differences between groups (Fig. S3A). Next, we tested short-term/acute intervention on S100A10 or S100A11 for potential beneficial effects on steatosis reduction in an early phase by downregulating for only 1 month either S100A10 or S100A11 at 4 months of age (Fig. 2I). In this protocol, neither the shS100A10 group nor the shS100A11 group presented improvement in lipid accumulation in the liver (Fig. 2J–M). This indicates the need for long-term downregulation of these proteins to observe alleviation of MASLD, although we cannot exclude that a 50% knock-down for one month (Fig. S3B) might not be enough to trigger beneficial effects.

Overall, this set of data shows for the first time the relevance of targeting S100A10 or S100A11, even after MASLD is established. This effect could participate to the attenuation of HCC development.

### Downregulation of S100A10 versus S100A11 trigger divergent outcomes on hepatocarcinogenesis in a MASLD-driven hepatic cancer model

**Fig. 3:**
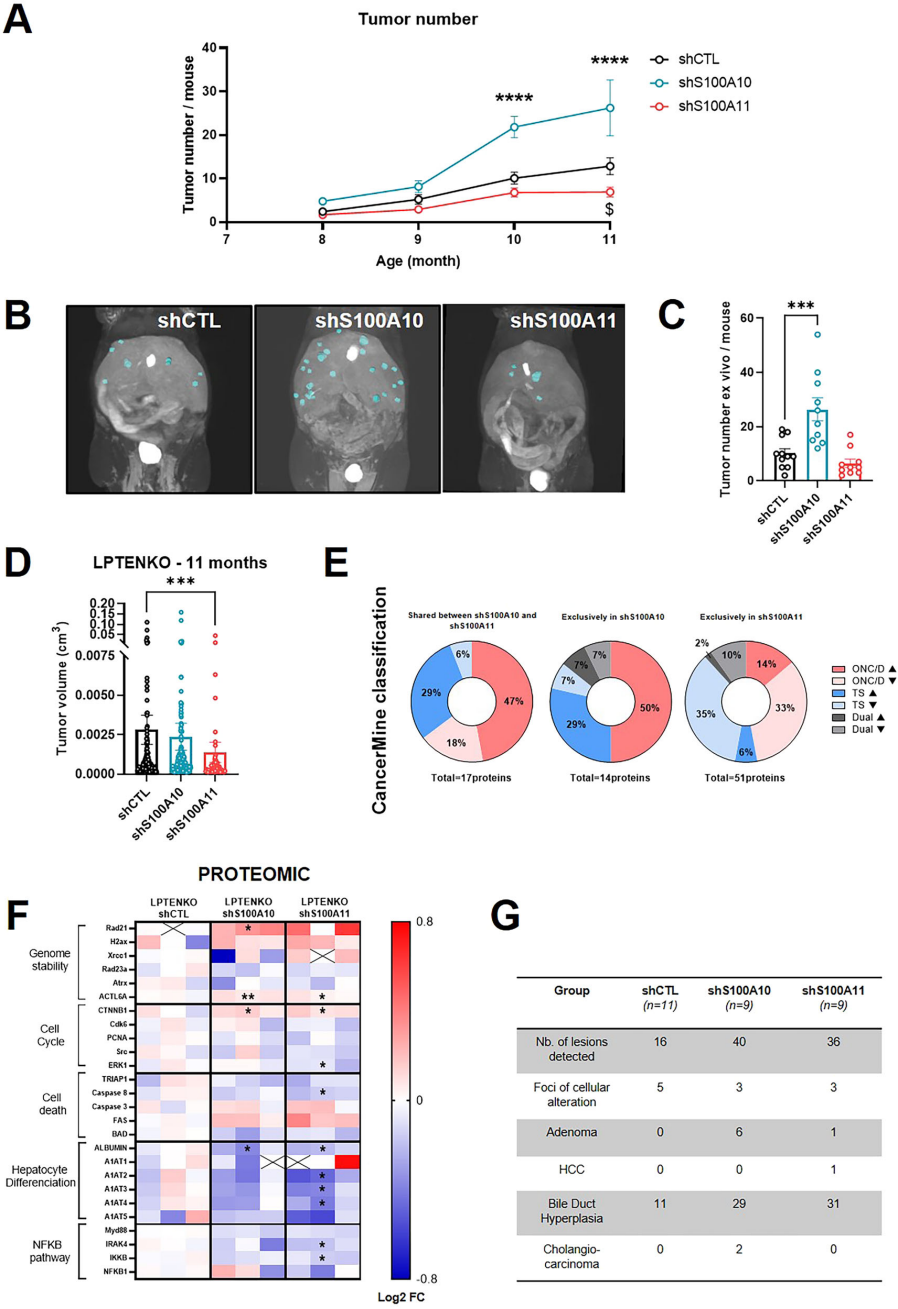
Downregulation of S100A10 versus S100A11 trigger divergent outcomes on hepatocarcinogenesis in a MASLD-driven hepatic cancer model. **A** MRI analysis of the tumor number per mouse in LPTENKO injected with AAV8-shCTL (*n* = 12–14), AAV8-shS100A10 (*n* = 9–13), or AAV8-shS100A11 (*n* = 11–13) at 8, 9, 10, and 11 months of age. **B** Representative 3D reconstruction of tumors in the liver of LPTENKO mice injected with AAV8-shCTL, AAV8-shS100A10, or AAV8-shS100A11 at 11 months of age. **C** Tumor count in the liver ex vivo, following dissection of the liver from LPTENKO mice injected with AAV8-shCTL (*n* = 11), AAV8-shS100A10 (*n* = 10), or AAV8-shS100A11 (*n* = 10) at 11 months of age. **D** Tumor volume measured by MRI analysis in LPTENKO mice injected with AAV8-shCTL (*n* = 149), AAV8-shS100A10 (*n* = 231), or AAV8-shS100A11 (*n* = 70) at 11 months of age. **E** CancerMine Classification of deregulated proteins shared between shS100A10 and shS100A11 group or exclusive to each group. ONC/D oncogenes/drivers, TS tumor suppressors, ▲ upregulated, ▼ downregulated. **F** Heatmap representation of proteomic analysis performed in the liver of 11 months-old LPTENKO injected with shCTL (*n* = 3), shS100A10 (*n* = 3), or shS100A11 (*n* = 3) for proteins involved in genome stability, cell cycle, cell death, hepatocyte differentiation, and the NF-kB pathway. Black crosses in white boxes represent non-detectable proteins in the sample. **G** Qualitative histopathological characterization of hepatocyte lesions (foci of cellular alteration, Adenoma or HCC) and biliary duct lesions (bile duct hyperplasia and cholangiocarcinoma) in 11 months LPTENKO-shCTL (*n* = 11), LPTENKO-shS100A10 (*n* = 9) or LPTENKO-shS100A11 (*n* = 9). Results are presented as means +/− S.E.M. “*n*” represents the number of animals or the number of tumors (**D**). * = *p* < 0.05; ** = *p* < 0.01; *** = *p* < 0.001; **** = *p* < 0.0001 determined by Two-Way ANOVA followed by Dunnett’s post-hoc analysis (**A**), by One-Way ANOVA followed by Dunnett’s post-hoc analysis (**C**), by Kruskall–Wallis test followed by Dunnett’s post-hoc analysis (**D**) or by *t*-test (**F**).

Tumor number incidence and size was monitored in LPTENKO mice with hepatocyte-specific knock-down of S100A10 or S100A11 through in vivo MRI imaging every month, in order to investigate the effect of S100A10/S100A11 downregulation on hepatic carcinogenesis in a MASLD context. Surprisingly, from the age of 8 months, LPTENKO-shS100A10 animals presented significantly more tumors than the LPTENKO-shCTL group (Figs. 3A–C and S4A). On the contrary, we observed a 50% reduction of tumor volumes in the animals knocked down for S100A11 (Figs. 3D and S4B). These findings suggest a critical role for S100A10 in hepatocarcinogenesis and for S100A11 in the tumoral growth. In order to identify possible oncogenes/drivers (ONC/D) or tumor suppressors (TS) modulated specifically by S100A10 or S100A11, we processed the proteomic data using CancerMine database (Fig. 3E and Table S1). Among the proteins known to have a role in cancer, 50% were classified as cancer drivers and upregulated in the shS100A10 group. Conversely, shS100A11 group presented a reduction of both drivers and tumor suppressors in the same extent. Besides, the proteomic showed a tendency for lower cell cycle components in the shS100A11 group, as well as the NF-κB pathway, highlighting S100A11’s role in the establishment of the inflammatory microenvironment (Fig. 3F). Genomic instability playing a major contribution in carcinogenesis we assessed this parameter by measuring γH2AX expression and showed no differences (Fig. S4C, D). In depth qualitative analysis of the type of lesions/histological alterations observed in LPTENKO liver sections upon S100A10 or S100A11 knock-down was performed by two expert board-certified clinical pathologists, as described by Thoolen and collaborators [23]. Their analysis revealed an increased incidence of adenomas in the shS100A10 group (Figs. 3G and S4E). Overall, only one HCC has been identified, in the shS100A11 group (HCC arise around 14–15 months in this model [14]). LPTENKO are known to exhibit biliary lesions [24]. Herein, the incidence of bile duct hyperplasia was increased upon S100A10 or S100A11 knock-down (Figs. 3G and S4E). Moreover, the only two cholangiocarcinomas identified were in the shS100A10 group of mice.

This set of data shows that S100A10 downregulation does not represent a suitable target to prevent hepatocarcinogenesis, but on the contrary, could be protective. Conversely, S100A11 downregulation appears to restrain tumor growth. The question remains if these outcomes rely on the MASLD context observed in LPTENKO mice.

### Downregulation of S100A10 versus S100A11 differently impact on tumoral initiation and growth, depending on the fatty liver context

**Fig. 4:**
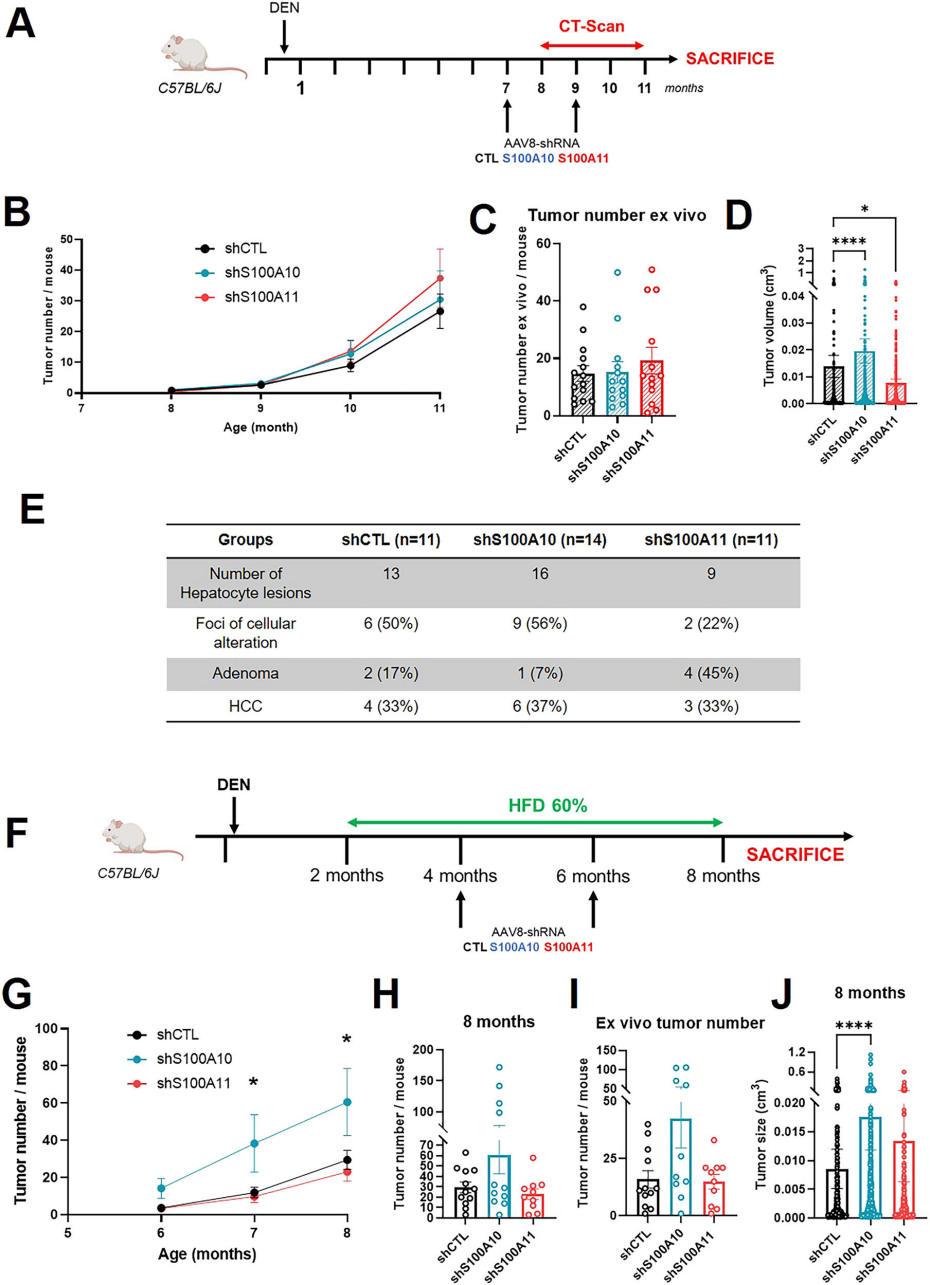
Downregulation of S100A10 versus S100A11 differently impact on tumoral initiation and growth, depending on the fatty liver context. **A** Experimental design of the diethylnitrosamine (DEN) protocol, including AAV8 injections, CT-scan measurements, and sacrifice. **B** CT-scan analysis of the tumor number per mouse in DEN mice injected with AAV8-shCTL (*n* = 12), AAV8-shS100A10 (*n* = 14), or AAV8-shS100A11 (*n* = 13) at 8, 9, 10, and 11 months of age. **C** Tumor counting in the liver ex vivo, following dissection of the liver from DEN mice injected with AAV8-shCTL (*n* = 14), AAV8-shS100A10 (*n* = 13), or AAV8-shS100A11 (*n* = 13) at 11 months of age. **D** Tumor volume measured using CT-scan analysis in DEN mice injected with AAV8-shCTL (*n* = 346), AAV8-shS100A10 (*n* = 425), or AAV8-shS100A11 (*n* = 486) at 11 months of age. **E** Qualitative histopathological characterization of hepatocyte lesions (foci of cellular alteration, Adenoma or HCC) in DEN mice injected with AAV8-shCTL (*n* = 11), AAV8-shS100A10 (*n* = 14) or AAV8-shS100A11 (*n* = 11). **F** Experimental design of the HFD-DEN protocol, including AAV8 injections, feeding period with High Fat Diet and euthanasia. **G** MRI analysis of the tumor number per mouse in HFD-DEN mice injected with AAV8-shCTL (*n* = 12), AAV8-shS100A10 (*n* = 11–12), or AAV8-shS100A11 (*n* = 10–13) at 6, 7, and 8 months of age. **H** Tumor number per mouse at 8 months-old assessed by MRI analysis in HFD-DEN mice injected with AAV8-shCTL (*n* = 12), AAV8-shS100A10 (*n* = 11), or AAV8-shS100A11 (*n* = 10). **I** Tumor counting in the liver ex vivo, following dissection of liver from HFD-DEN mice injected with AAV8-shCTL (*n* = 12), AAV8-shS100A10 (*n* = 10), or AAV8-shS100A11 (*n* = 10), at 8 months of age. **J** Tumor volume measured using CT-scan analysis in DEN mice injected with AAV8-shCTL (*n* = 353), AAV8-shS100A10 (*n* = 664), or AAV8-shS100A11 (*n* = 230), at 11 months of age. Results are presented as means +/− S.E.M. “*n*” represents the number of animals or the number of tumors (**D** and **J**). * = *p* < 0.05; **** = *p* < 0.0001 determined by Two-Way ANOVA followed by Dunnett’s post-hoc analysis (**B** and **G**), by One-Way ANOVA followed by Dunnett’s post-hoc analysis (**H**, **I**), or by Kruskall–Wallis test followed by Dunnett’s post-hoc analysis (**C**, **D**, and **J**) or by *t*-test (Fig. 4G).

Since etiology of liver cancer is multifactorial and multigenic, we next investigated the respective roles of S100A10 and S100A11 in an additional mouse model with or without the contribution of MASLD. In this model, a single injection of DEN triggers DNA damages and multiplication of mutations within the genome [25]. In DEN-treated mice, S100A10 and S100A11 were downregulated at 7 months (before tumor formation) up to 11 months of age (time of sacrifice) (Fig. 4A). The knock-down of S100A10 or S100A11 did not affect the number of tumors induced by the DEN (Figs. 4B, C and S5A). However, this model once again revealed divergent effects of these two S100 proteins in tumoral growth (Figs. 4D and S5B). While the knock-down of S100A10 resulted in increased size of the nodules, S100A11 downregulation reduced tumor growth, consistent with the LPTENKO model, underscoring a MASLD-independent effect. The downregulation of S100 proteins did not affect significantly the incidence rates of different type of hepatic lesions (i.e., foci of cellular alteration, adenoma, or HCC). The knock-down of S100A11 seems to promote the occurrence of adenomas (Fig. 4E), but the number of lesions being analyzed was limited.

We hypothesized that the protective role of S100A10 on hepatocarcinogenesis could depend on whether it is occurring in a MASLD context or not. Therefore, we induced steatosis and obesity (Fig. S6A) in DEN-injected mice by feeding them with a 60% high-fat diet (HFD) from 2 months of age to 8 months (Fig. 4F). The S100A10 and S100A11 shRNAs did not modify body weight gain over 6 months (Fig. S6B). Under HFD conditions, the shS100A10 group presented higher numbers and volume of tumors compared to the control group (Figs. 4G–J and S6C, D). On the contrary, the shS100A11 group did not show any differences regarding growth/number of tumors.

Based on both the DEN/DEN-HFD and the LPTENKO models, we identified a crucial protective role for S100A10 on tumoral initiation, in MASLD setting, whereas S100A11 favors growth of tumors in two out of 3 models analyzed.

### S100A10 upregulation in the liver reduces tumoral burden in a mouse model mimicking human β-catenin mutated subtype of HCC

**Fig. 5:**
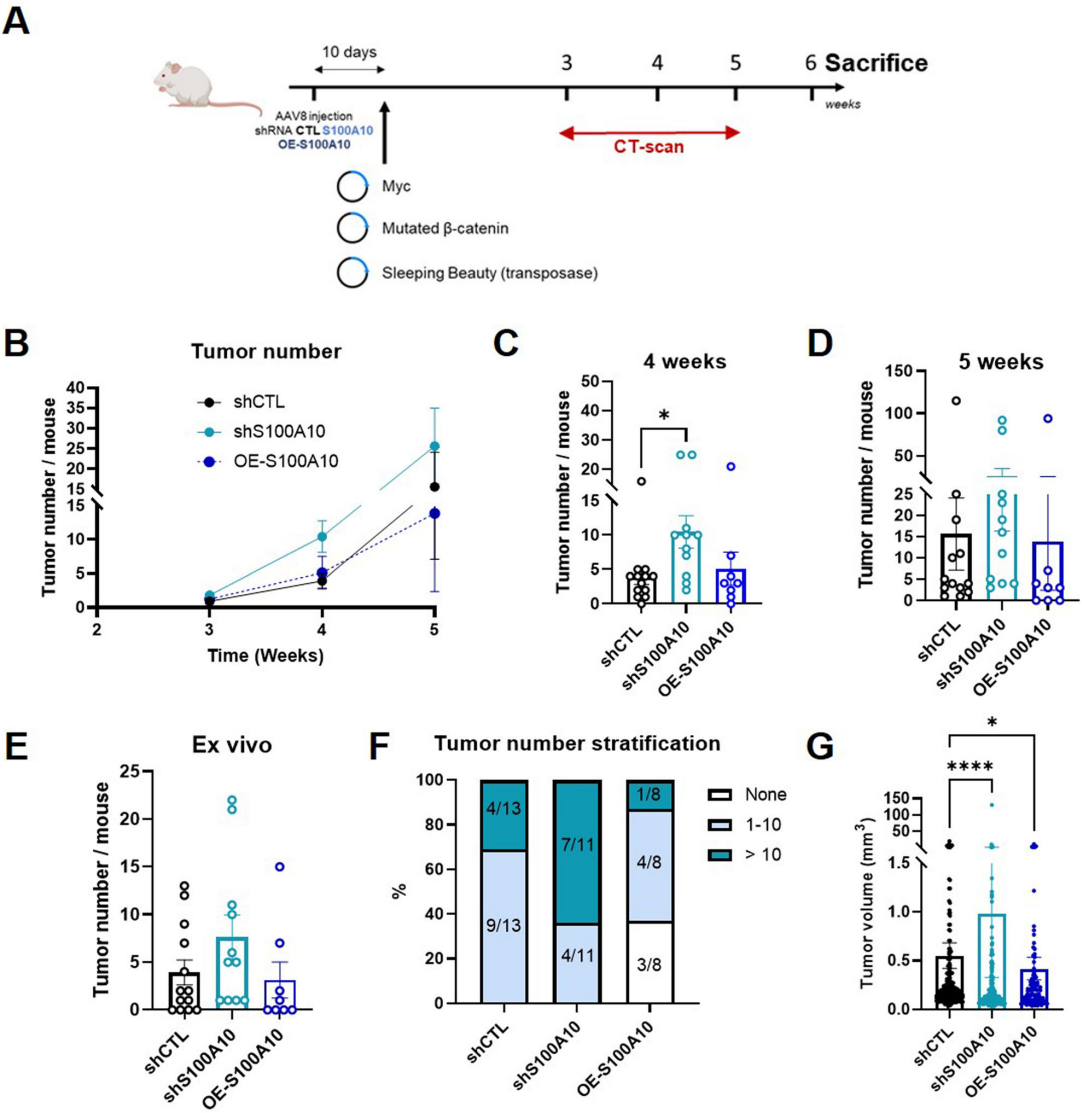
S100A10 upregulation in the liver reduces tumoral burden in the mouse model mimicking human β-catenin mutated subtype of HCC. **A** Experimental design, including AAV8 injections, hydrodynamic injections of plasmids encoding human form of Myc, human constitutively active β-catenin and SB transposase, CT-scan measurements and the sacrifice. **B** CT-scan analysis of tumor number per mouse in Myc/mutated β-catenin overexpression mouse model injected with AAV8-shCTL (*n* = 13), AAV8-shS100A10 (*n* = 11), or AAV8-OE-S100A10 (*n* = 8) at 3, 4, and 5 weeks post-hydrodynamic injection. **C** CT-scan analysis of tumor number per mice in Myc/mutated β-catenin overexpression mouse model injected with AAV8-shCTL (*n* = 13), AAV8-shS100A10 (*n* = 11), or AAV8-OE-S100A10 (*n* = 8) 4 weeks after hydrodynamic injection. **D** CT-scan analysis of tumor number per mouse in Myc/mutated β-catenin overexpression mouse model injected with AAV8-shCTL (*n* = 13), AAV8-shS100A10 (*n* = 11), or AAV8-OE-S100A10 (*n* = 8) 5 weeks after hydrodynamic injection. **E** Tumor counting in the liver ex vivo, following dissection of Myc/mutated β-catenin overexpression mouse model injected with AAV8-shCTL (*n* = 13), AAV8-shS100A10 (*n* = 11) or AAV8-OE-S100A10 (*n* = 8) 6 weeks after hydrodynamic injection. **F** Tumor number stratification in Myc / mutated β-catenin overexpression mouse model injected with AAV8-shCTL (*n* = 13), AAV8-shS100A10 (*n* = 11) or AAV8-OE-S100A10 (*n* = 8), analyzed 5 weeks after hydrodynamic injection. **G** Tumor volume measured by CT-scan analysis in Myc/mutated β-catenin overexpression mouse model injected with AAV8-shCTL (*n* = 200), AAV8-shS100A10 (*n* = 201), or AAV8-OE-S100A10 (*n* = 111) 5 weeks after hydrodynamic injection. Results are presented as means +/− S.E.M. “*n*” represents the number of animals or the number of tumors (**F**). * = *p* < 0.05; **** = *p* < 0.0001 determined by Two-Way ANOVA followed by Dunnett’s post-hoc analysis (**B**), by One-Way ANOVA followed by Dunnett’s post-hoc analysis (**D** and **E**), or by Kruskall–Wallis test followed by Dunnett’s post-hoc analysis (**C** and **G**).

As our data unprecedently reveal potential hepatoprotective role for endogenous S100A10, we then over-expressed S100A10, and assessed tumor initiation and cancer progression. By doing so, we are mimicking the increased levels of S100A10 observed in human HCC, before tumor formation. For this aim, we used a well-established HCC mouse model for a specific subtype of human HCC (over-expressing Myc and a mutated form of the β-catenin leading to its constitutive activation) [26]. Although not displaying hepatic steatosis, the tumors developing in this setting are known to be lipophagic [16], which could be relevant for the protective effects of S100A10. In these mice, we either downregulated or over-expressed S100A10 by AAV8 injection (Fig. 5A). At 4 weeks post-transduction, downregulation of S100A10 resulted in increased number of tumors as revealed by CT-scan, but in a lower extent as compared to LPTENKO or HFD-DEN models (Fig. 5B, C). At 5 weeks, significance was lost in term of tumor number, even though 64% of the shS100A10 mice had more than 10 tumors versus 31% for the shCTL group (Fig. 5B–F). Tumor volume was significantly increased in shS100A10 mice (Fig. 5G). Overexpression of S100A10 did not significantly impact on the number of tumors, nevertheless the only 3 mice that did not develop any tumors were in the OE-S100A10 group. In the same regard, only 1 mouse had more than 10 tumors (Fig. 5B–F). Of interest, tumor size was significantly reduced in the OE-S100A10 group (Fig. 5G).

Based on these data, preventive S100A10 over-expression restrains tumor growth and might protect against liver tumor incidence.

### Long-term S100A10 protein stability in the liver depends on the presence of S100A11 in vivo

**Fig. 6:**
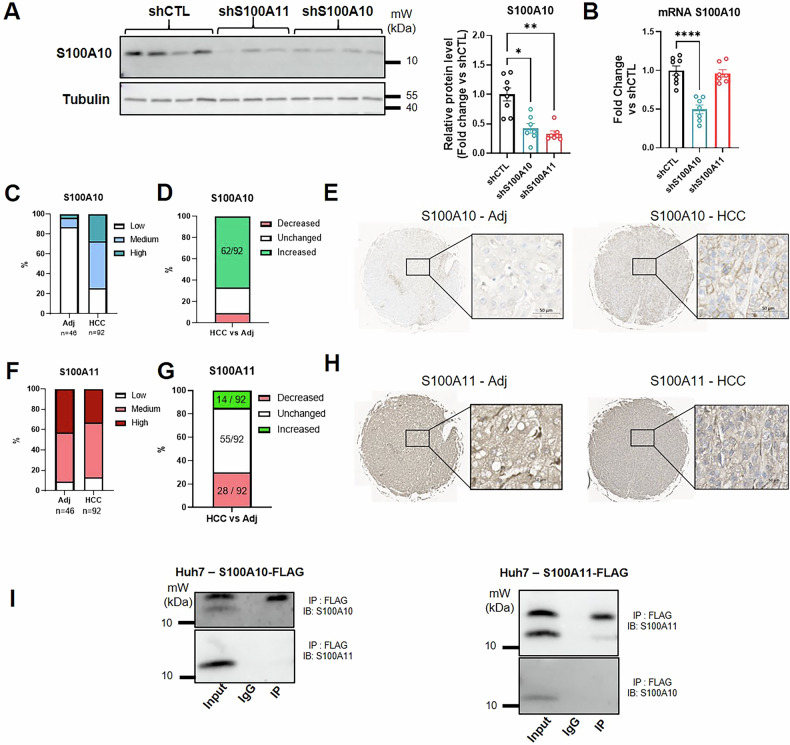
Long-term S100A10 protein stability in the liver depends on the presence of S100A11 in vivo. **A** Relative protein level of S100A10 measured by western-blot in the liver of 5 months-old LPTENKO injected at 4 months with AAV8-shCTL (*n* = 8), AAV8-shS100A10 (*n* = 7), or AAV8-shS100A11 (*n* = 7). Tubulin was used as housekeeping protein. Results are expressed as fold change versus the shCTL group. **B** Relative mRNA level of S100A10 measured by qPCR in the liver of 5 months-old LPTENKO injected at 4 months with AAV8-shCTL (*n* = 8), AAV8-shS100A10 (*n* = 7), or AAV8-shS100A11 (*n* = 7). Cyclophilin A was used as housekeeping gene. Results are expressed as fold-change versus the shCTL group. **C** Scoring of S100A10 immunohistochemistry performed on liver TMA from HCC patients comparing HCC (*n* = 92, two per patient) and adjacent non-tumoral tissue (*n* = 46, one per patient). Results are expressed as the percentage of low, medium, or high intensity of the staining. **D** Scoring of S100A10 immunohistochemistry performed on liver TMA from HCC patients comparing the staining intensity in the tumoral compartment versus the non-tumoral part for every patient. Results are expressed as the percentage of decreased (red), unchanged (white), or increased (green) staining intensity in the tumor in comparison to the non-tumoral liver. **E** Representative images of S100A10 staining (brown) in adjacent non-tumoral liver (left) and in HCC (right). Nuclei are counterstained with hematoxylin. Scale bar: 50 µm. **F** Scoring of S100A11 immunohistochemistry performed on liver TMA from HCC patients comparing HCC (*n* = 92, two per patient) and adjacent non-tumoral tissue (*n* = 46, one per patient). Results are expressed as the percentage of low, medium, or high intensity of the staining. **G** Scoring of S100A11 immunohistochemistry performed on liver TMA from HCC patients comparing the staining intensity in the tumoral compartment versus the non-tumoral part for every patient. Results are expressed as the percentage of decreased (red), unchanged (white), or increased (green) staining intensity in the tumor in comparison to the non-tumoral liver. **H** Representative images of S100A11 staining (brown) in adjacent non-tumoral liver (left) and in HCC (right). Nuclei are counterstained with hemalum. Scale bar: 50 µm. **I** Western blot analysis of S100A10 and S100A11 following FLAG immunoprecipitation of the cell lysates from Huh7 cells transfected with S100A10-FLAG (upper panel) or S100A11-FLAG (lower panel). Results are presented as means +/− S.E.M. “*n*” represents the number of animals (**A**, **B**, and **J**), the number of human liver biopsies (**C**, **D**, **F**, and **G**). * = *p* < 0.05; ** = *p* < 0.01; *** = *p* < 0.001 determined by One-Way ANOVA followed by Dunnett’s post-hoc analysis (**A**, **B**).

The present work shows that silencing S100A10 or S100A11 resulted in distinct outcomes in hepatic malignant transformation and cancer progression. The reasons why these two related proteins exhibit such opposite effects are unclear. Surprisingly, we observed that hepatic S100A11 downregulation decreased S100A10 protein level (1 month post-AAV8, Fig. 6A), even though the corresponding mRNA levels were preserved (Fig. 6B). This effect was also observed in a longer timeframe through proteomic analysis (5 months post-AAV8 injection, Fig. S7A). In order to check if protein levels of S100A10 and S100A11 correlated also in human HCC, we analyzed Tissue-Micro Array (TMA) of 46 HCC patients (2 tumors per patient) and 3 Intra-Hepatic Cholangiocarcinoma (IHC) patients versus non-tumoral part of the liver. The intensity of the two staining was scored as low, medium or high and compared to the intensity of the corresponding adjacent liver tissue. While S100A10 protein levels were significantly increased in HCC or IHC in comparison to non-tumoral hepatic tissue (Figs. 6C–E and S7B), this was observed in only 15% of the tumors for S100A11 (Figs. 6F–H and S7C–E). Since grade 1, S100A10 staining was stronger than in adjacent non-tumoral part (Fig. S7F), highlighting S100A10 as an early biomarker of HCC in liver biopsies. Gender did not influence these observations (Fig. S7G). Interestingly, in the tumoral compartment, there was an enrichment of S100A10 at the plasma membrane, whereas in the non-tumoral part, S100A10 was mainly in the cytoplasm (Figs. 6E, S7H, and S8A). This membrane enrichment of S100A10 was not observed in commonly used hepatoma cells lines (Fig. S8B) underscoring differences in protein localization that could explain potential discrepant data on S100A10 in these in vitro systems. The localization of S100A11 in the hepatocytes remained mostly in the cytoplasm/nucleus during carcinogenesis showing that S100A10 and S100A11 were not in the same compartment during the disease development. This was supported by co-immunoprecipitation experiments in Huh7 cells showing no interaction between S100A10 and S100A11, at least in this in vitro system (Fig. 6I). Therefore, the instability of S100A10 upon S100A11 downregulation might be the consequence of a long-term compensation in order to maintain a balance rather than a missing interaction between these two proteins as reported for S100A8 and S100A9 [27]. Accordingly, downregulation of S100A11 for 3 days did not alter the protein levels of S100A10 in mouse primary hepatocytes, nor in Huh7 cells (Fig. S9A, B), highlighting once again a long-term compensatory effect in vivo.

Overall, in vivo preservation of S100A10 levels rely on the presence of S100A11 for its protein stability rather than for its transcription.

## Discussion

MASLD and HCC are major liver diseases affecting the global population. Currently available therapeutic strategies are sub-optimal, calling for novel biomarkers and molecular targets in order to improve diagnostic and restrain the pandemic spread of these liver disorders. In the past few years, S100 proteins were included in the protein network dysregulated upon MASLD progression and HCC development. Indeed, their upregulation is considered as potential biomarker of simple steatosis, MASH, and HCC [6]. To the best of our knowledge, all the S100 proteins thus far investigated showed liver pro-oncogenic properties. More precisely, exosome-derived S100A10 fosters HCC growth in xenograft models [7], while S100A4 favors metastasis [28], and macrophage-derived S100A9 promotes stemness [29]. Moreover, their over-expression observed in HCC patients [6] supported the hypothesis that S100 proteins favor hepatocarcinogenesis. Among them, high expression of S100A10 and S100A11 have been associated with low survival in HCC patients [7, 8]. These correlative clinical studies can hardly recapitulate what happens during the progression of the disease regarding the requirement of S100A10 and S100A11 in the development of tumors, which can be addressed using mouse models. Since liver cancer development is multifactorial and multigenic, we decided to use several complementary mouse models in order to recapitulate the complexity of the human disease.

The present results show for the first time an unexpected protective role for S100A10 in hepatic tumor incidence and progression, in addition to the one previously reported in ulcerative colitis [30], while contributing to MASLD. Indeed, targeting S100A10 with AAV-based strategy reduced the extent of steatosis and fibrosis. However, the strategy needs a long-term inhibition, as 1 month of downregulation was not sufficient to decrease the levels of triglycerides stored in the hepatocytes in the LPTENKO model. Mechanistically, steatosis might have been reduced by an increase of either VLDL production/secretion and fatty acid degradation concomitantly to a decreased of de novo lipogenesis. Regarding the role of S100A10 in fibrogenesis, nothing is known, except that its interaction with CFTR could play a role in lung fibrosis upon cystic fibrosis [31]. In our study, we show a beneficial effect of hepatocyte-specific S100A10 knock-down in collagen deposition in a mouse model of MASH. Whether this effect is a consequence of an improvement of steatosis or a direct contribution of S100A10 on stellate cells activation remains to be established.

Regarding the potential of targeting S100A11 to treat MASLD/MASH, S100A11 knock-down exhibited similar outcomes as S100A10 downregulation, but with more potent effects. Indeed, as previously shown in other studies performed in high fat diet-induced MASLD models [10, 11], preventive targeting S100A11 reduces steatosis. Here, we observed a beneficial effect once the disease is already ongoing. S100A11 has been shown to participate to myocardial [32] and chemically-induced liver fibrosis [9] through TGF-β dependent signaling pathways. In our study, the downregulation of S100A11 completely abrogated intra-sinusoidal collagen deposition. This suggests that either S100A11 represents the strongest promotor of steatosis/fibrosis among the two proteins or that the positive outcomes observed in shS100A11 groups are partially due to concomitant S100A10 protein downregulation; two hypotheses not mutually exclusive. When comparing the common biological processes deregulated by the silencing of S100A10 or S100A11, we highlighted the dynamic of fatty acid synthesis/degradation, which might be the shared function between these two proteins.

One of the most striking novelties in this study is the protective role of S100A10 on hepatocarcinogenesis and tumoral growth, besides the involvement of this protein in MASLD. Indeed, in the multiple mouse models we used showing diverse etiologies (LPTENKO, HFD-DEN, and Myc/mutated β-catenin over-expression), the knock-down of S100A10 consistently increased the number of nodules. We did not observe that phenomenon in DEN-injected mice devoid of the MASLD context. Therefore, we hypothesize that S100A10 protects against liver tumor incidence specifically in a context of steatotic liver disease (LPTENKO and HFD-DEN) or in lipophagic, less aggressive tumors (mutated β-catenin tumors [16]) representing by itself around 30% of HCC patients. This points to an intriguing paradox because S100A10 would contribute to MASLD and, at the same time, protects from further evolution toward HCC, underscoring two distinct functions of this protein. Translated at the clinical level, one can hypothesize that activation of S100A10 in patients presenting steatosis might dampen cancer initiation/progression. S100A10 has been widely described to play a key role in the brain in the context of depression, seizure, and neurodegenerative diseases [33]. From this perspective, the systemic of delivery of modulators of S100A10 should not pass the blood-brain barrier. Moreover, S100A10 presents central physiological roles in endothelial cell function and coagulation processes that could require a liver-specific targeting strategy in order to reduce the off-target effects, or would necessitate a partial modulation.

The outcome of our study regarding S100A10’s effect on tumoral growth is in disagreement with other studies performed in vitro where a deleterious role of S100A10 was pinpointed in cancer hepatocyte cell lines [13, 34] as well as in other cancer forms (reviewed in [35]). This highlights important discrepancies between in vitro and in vivo models, that may rely on the tumor microenvironment as described in liver and lung cancers [36, 37]. In addition, in a study performed by Wang and collaborators [7], exosome-derived S100A10 was shown to be detrimental by stimulating the stemness of hepatocytes as well as by enhancing metastasis development. This pushes the reasoning toward a dual action between S100A10 fraction present at the membrane of exosomes and intracellular S100A10. In our study, we observed membrane localization of S100A10 in HCC. If this accumulation drives the tumor suppressive properties of S100A10 remains unexplored and requires further investigations. Overall, these contradictory data might be explained by the fact that, in comparison to all these other works, S100A10 function was investigated in an in vivo context in our study, and not in xenograft approaches that do not recapitulate the early steps of hepatocarcinogenesis nor the tumor microenvironment. Moreover, the protective role of S100A10 was uncovered essentially when the liver of mice was steatotic. Based on the literature [6] and our own data, S100A10 protein level is increased in HCC. As we demonstrate an aggravation of hepatocarcinogenesis upon S100A10 downregulation, we can argue that its overexpression may act as a tumor suppressive mechanism of the cell to restrain tumor development. From this perspective, we decided to over-express S100A10 to mitigate liver cancer incidence/growth. Although it did not affect significantly the number of tumors, it significantly reduces tumor volume, showing positive outcomes in regards to cancer progression. More work has to be performed in the utilization of S100A10 as a possible tool to be used in the clinical management of HCC. For instance, we can imagine to couple S100A10 delivery with lower doses of clinically recommended chemotherapeutic drugs (e.g., sorafenib) to further assist in reducing the tumoral burden. Regarding the possible mechanisms explaining the role of S100A10 in hepatocarcinogenesis, our proteomic approach highlighted some interesting candidates. Among them, the upregulation of the anti-apoptotic factor Bcl2L1 and the malignant-driver RhoC need further investigations.

One of the main S100 protein associated with MASLD risk and low-survival in HCC is S100A11 [8, 10, 38]. Therefore, S100A11 is believed to be one of the most targetable members of this family in the management of MASLD and HCC. In light of this, we found that S100A11 silencing resolved MASLD development in a chronic stage, and slowed down tumor growth without affecting the number of nodules. However, targeting S100A11 downregulation to attenuate liver tumor incidence, although potentially promising approach, it also leads to the downregulation of the hepatoprotective S100A10, which might explain the absence of beneficial outcome on tumor incidence. Alternatively, the use of pharmacological inhibitors of S100A11 might preserve S100A10 protein, which needs to be further assessed. In accordance with previously published work based on the loss of PCK1 [39], we observed in various preclinical in vivo models of HCC a striking effect of S100A11-targeting strategy on slowing-down tumoral growth. This might be the consequence of the reduction of EGFR, MIEN1, or Transgelin-2 identified by our proteomic and described to foster tumor development [40–42], and/or the result of enhanced necroptosis (12 deregulated proteins identified by proteomic), S100A11 having recently been identified as a necroptosis-related gene signature in HCC [43].

Our results, shed light on a complex network of regulations upon MASLD-HCC, pointing to S100A10 and S100A11 interplay. To our knowledge, no direct interaction between these two proteins has been observed, as documented hereby in cultured hepatocytes, as opposed to the previously reported regulation of S100A8 and S100A9 heterodimer [27]. Annexin A2 (ANXA2) might represent the missing link of this uncharacterized regulation, as ANXA2 can bind both S100A10 and S100A11 [35, 44]. In concert with S100A11, ANXA2 is described as a driver of liver cancer [8, 45] and S100A10 controls the activity of ANXA2 [46]. One could hypothesize that, when present, S100A10 traps ANXA2, thereby slowing down its interaction with S100A11 and protecting against HCC progression. However, when S100A10 is lost, or decreased, ANXA2 would be more accessible to trigger liver carcinogenesis.

Finally, an important point of discussion is the methodology used to target S100A10 and S100A11. AAVs are widely used in research based on their hepatotropic properties. The utilization of shRNAs to downregulate either S100A10 or S100A11 led to a strong knock-down in the first weeks and around 50% at 1 to 3 months post-injection, requiring a second injection of AAV8. Consequently, the effect reported in this study, with long-standing protocols, should be considered as rather drastic since about 50% of the targeted protein was left in the tissue. Thus, it raises hope for the utilization of pharmacological inhibitors of these proteins, which often do not fully abrogate the function of their target.

Overall, our study highlighted for the first time an hepatoprotective contribution of S100A10 on liver cancer. In parallel, we clarified the beneficial outcomes of targeting S100A11 in multiple pre-clinical models of MASLD and hepatic tumorigenesis. This might open new avenues regarding the utilization of S100 proteins as therapeutic targets in the management of liver disorders such as MASLD and HCC.

## Supplementary information

Supplemental Figure 1

Supplemental Figure 2

Supplemental Figure 3

Supplemental Figure 4

Supplemental Figure 5

Supplemental Figure 6

Supplemental Figure 7

Supplemental Figure 8

Supplemental Figure 9

Supplemental Table 1

Supplemental Material and Methods

Supplemental uncropped western blot

## Data Availability

Data presented in this manuscript will be publicly available on 10.26037/yareta:msbz7hw3fzc2rpjiqinyqs4jtq.
