# Supplementary figures and images for: Comparative analysis of S100A10 and S100A11 in MASLD and hepatic cancer development revealed a tumor suppressive role for S100A10
Source: Cell Death Dis. 2025 Aug 21;16(1):633. doi: 10.1038/s41419-025-07940-2 (PMC12370981; doi:10.1038/s41419-025-07940-2)

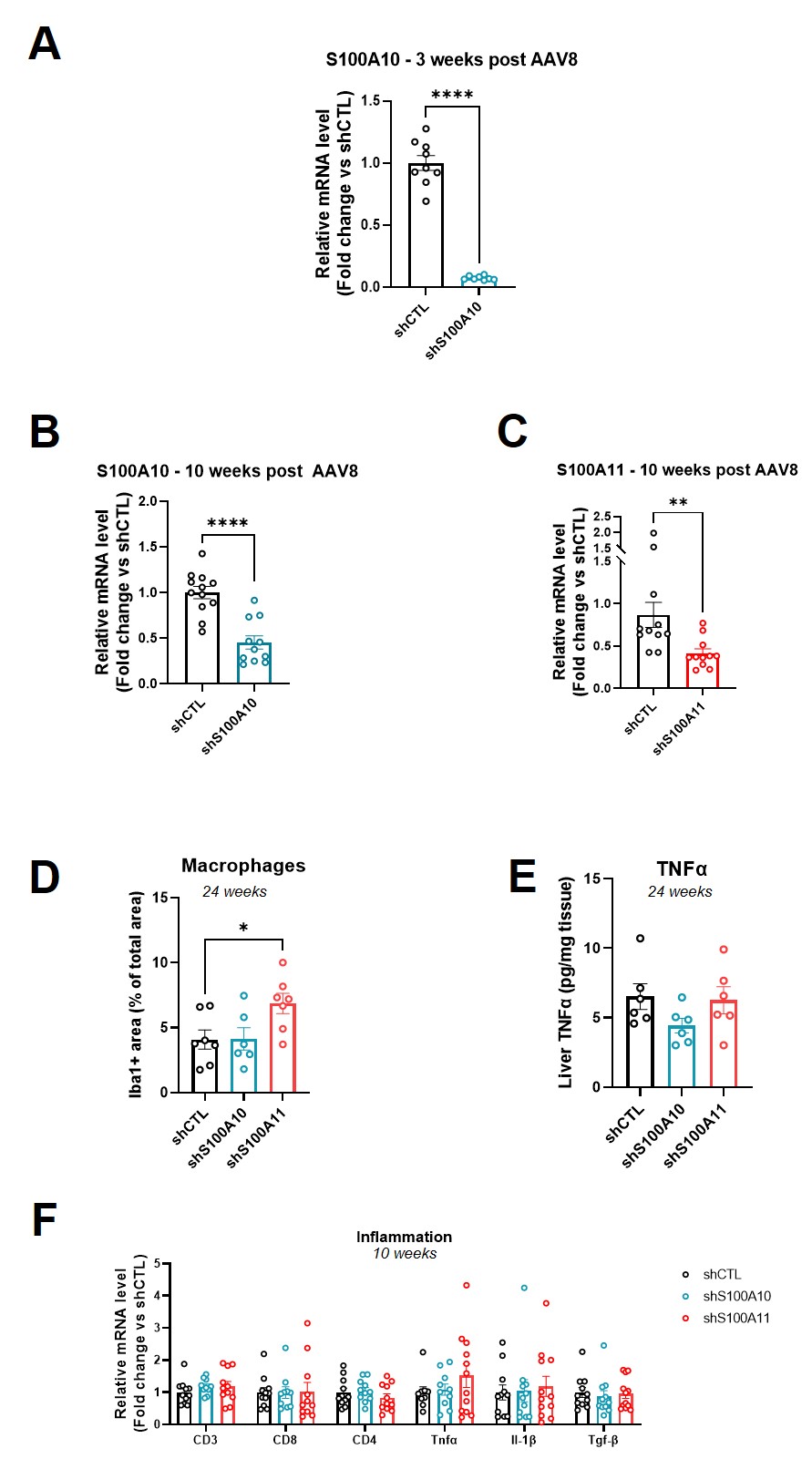

Supplement: Supplementary file 1 — Supplemental Figure 1 [file 41419_2025_7940_MOESM1_ESM.jpg]

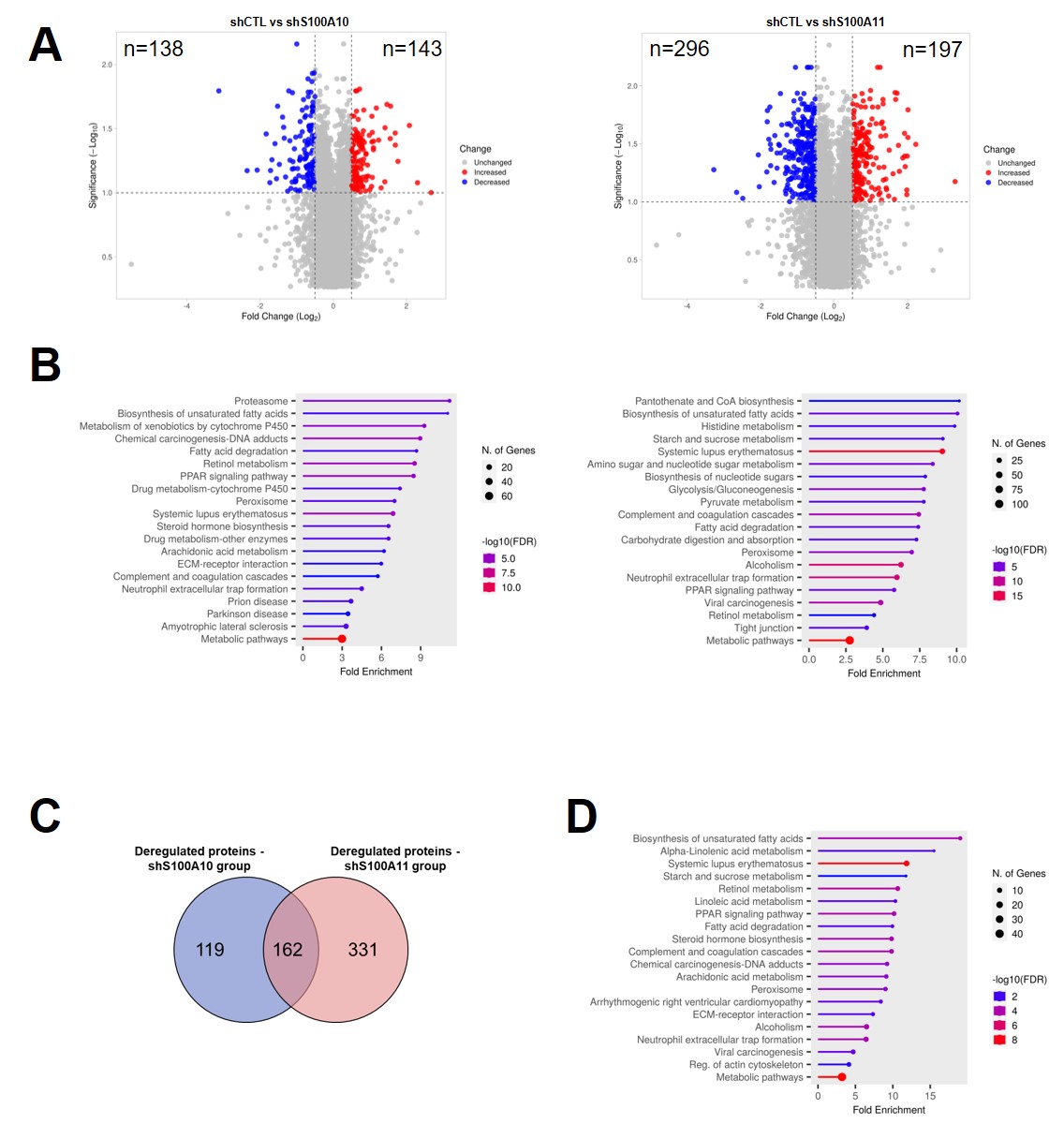

Supplement: Supplementary file 2 — Supplemental Figure 2 [file 41419_2025_7940_MOESM2_ESM.jpg]

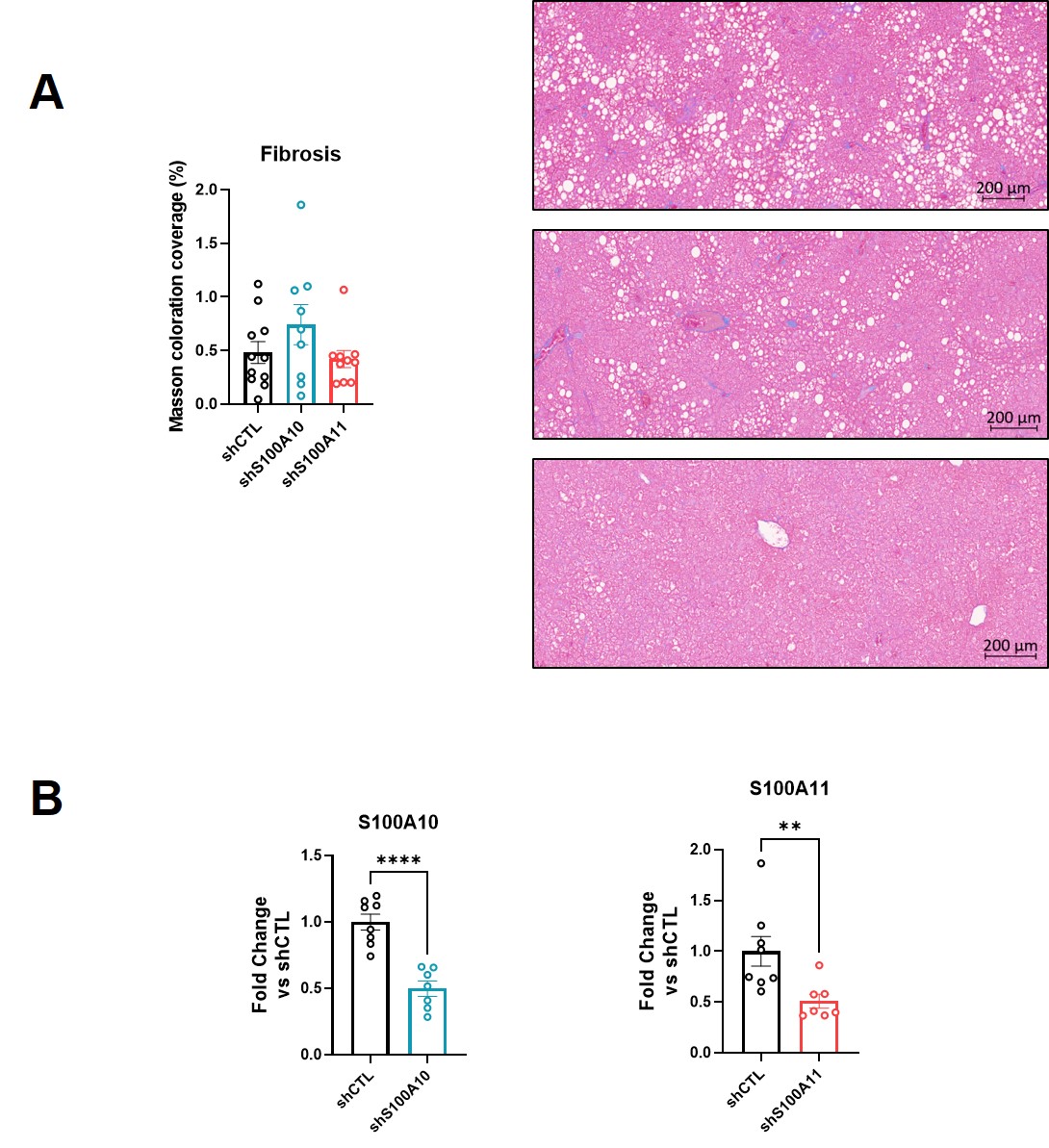

Supplement: Supplementary file 3 — Supplemental Figure 3 [file 41419_2025_7940_MOESM3_ESM.jpg]

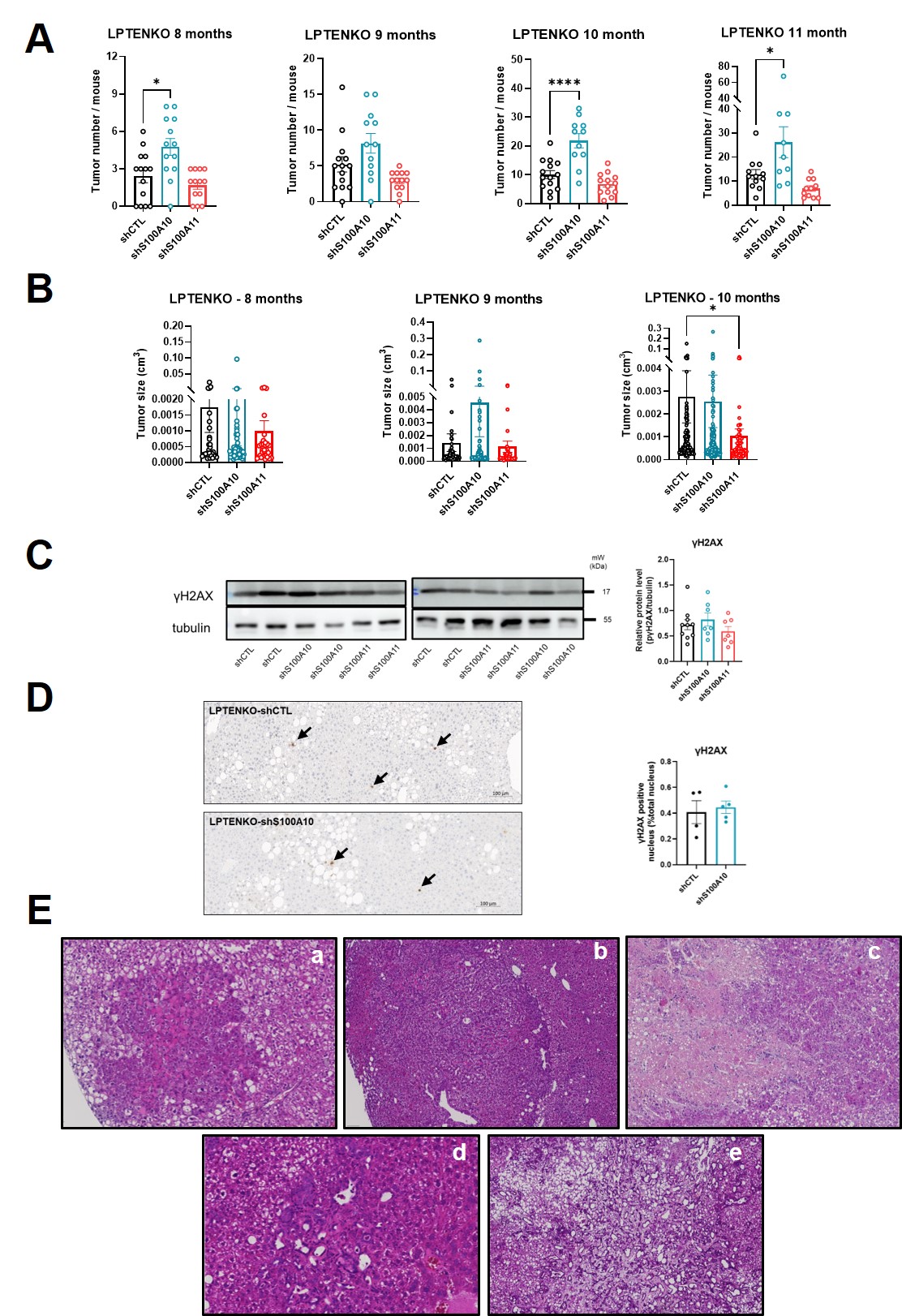

Supplement: Supplementary file 4 — Supplemental Figure 4 [file 41419_2025_7940_MOESM4_ESM.jpg]

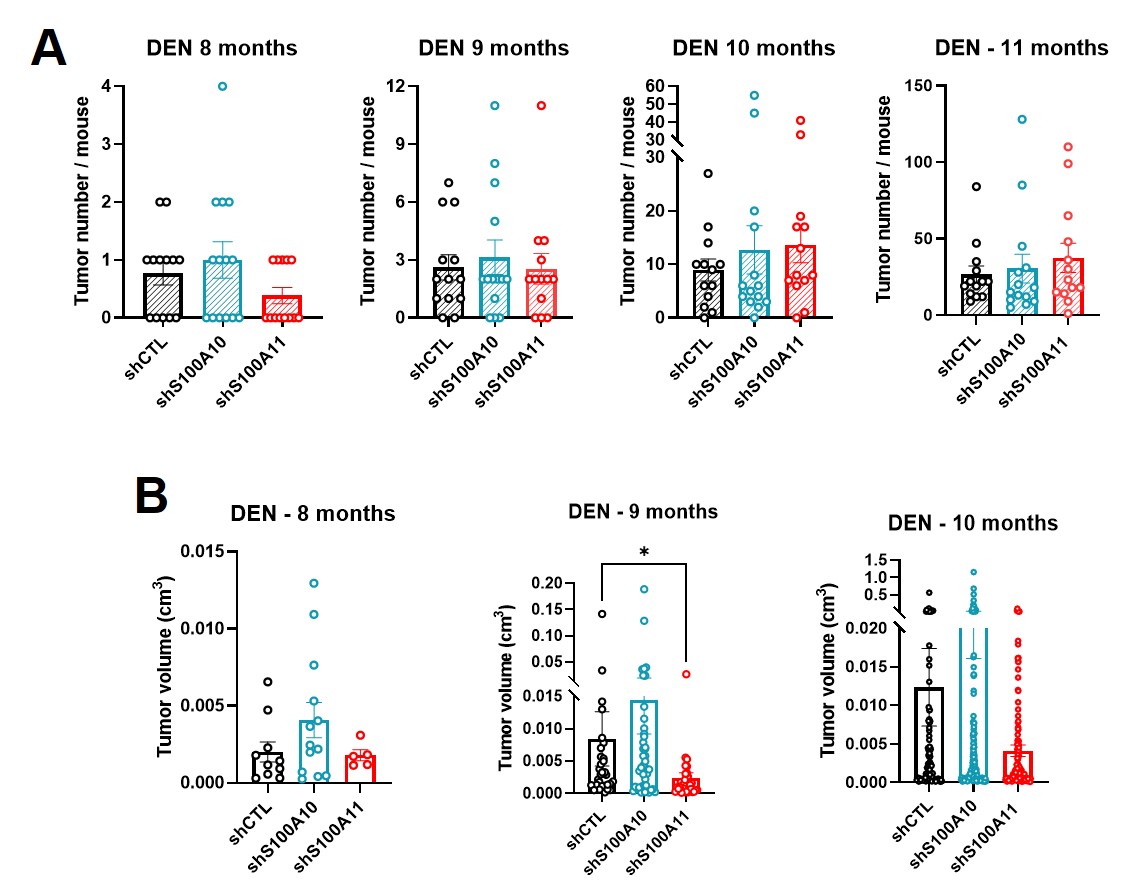

Supplement: Supplementary file 5 — Supplemental Figure 5 [file 41419_2025_7940_MOESM5_ESM.jpg]

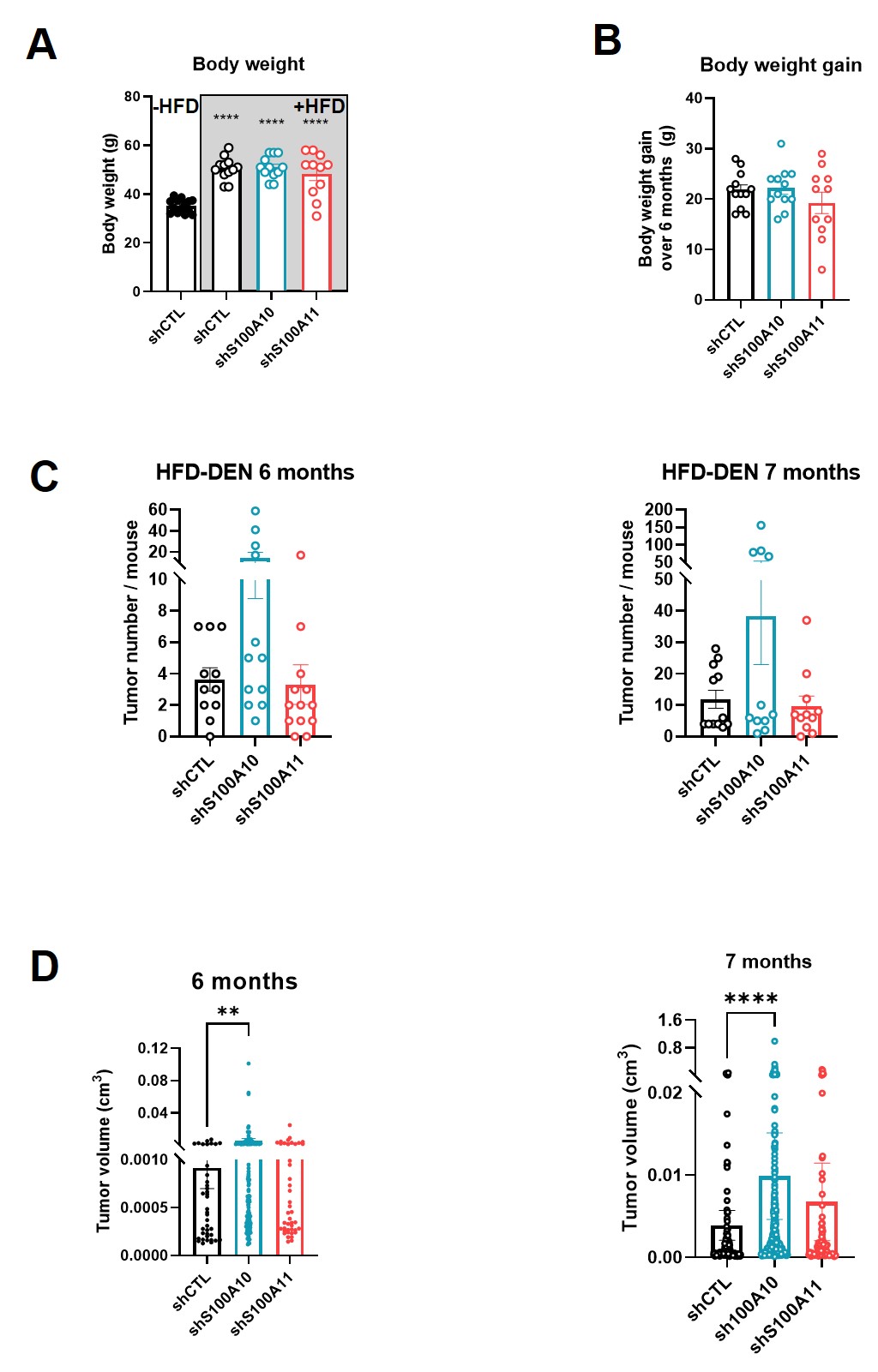

Supplement: Supplementary file 6 — Supplemental Figure 6 [file 41419_2025_7940_MOESM6_ESM.jpg]

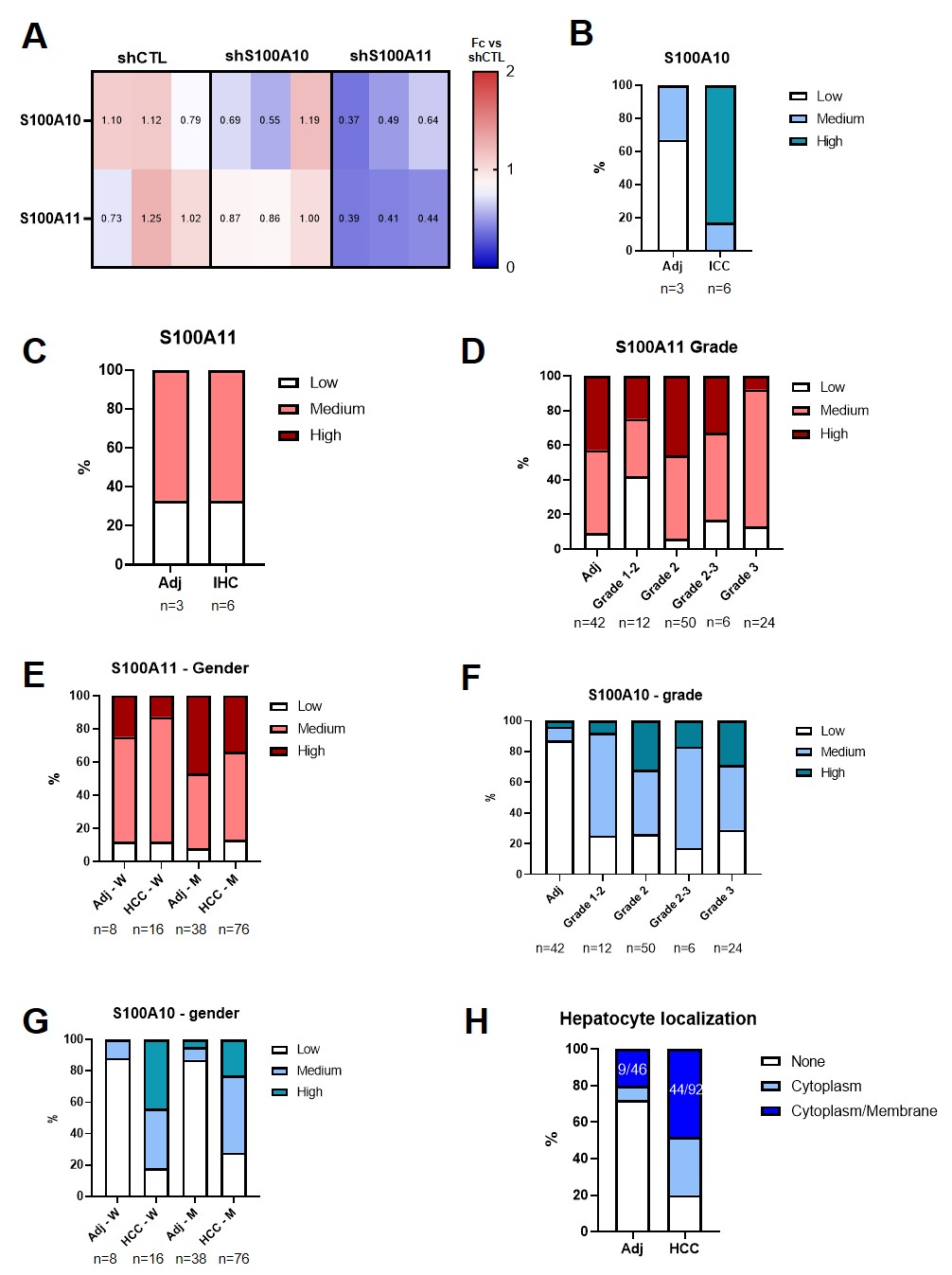

Supplement: Supplementary file 7 — Supplemental Figure 7 [file 41419_2025_7940_MOESM7_ESM.jpg]

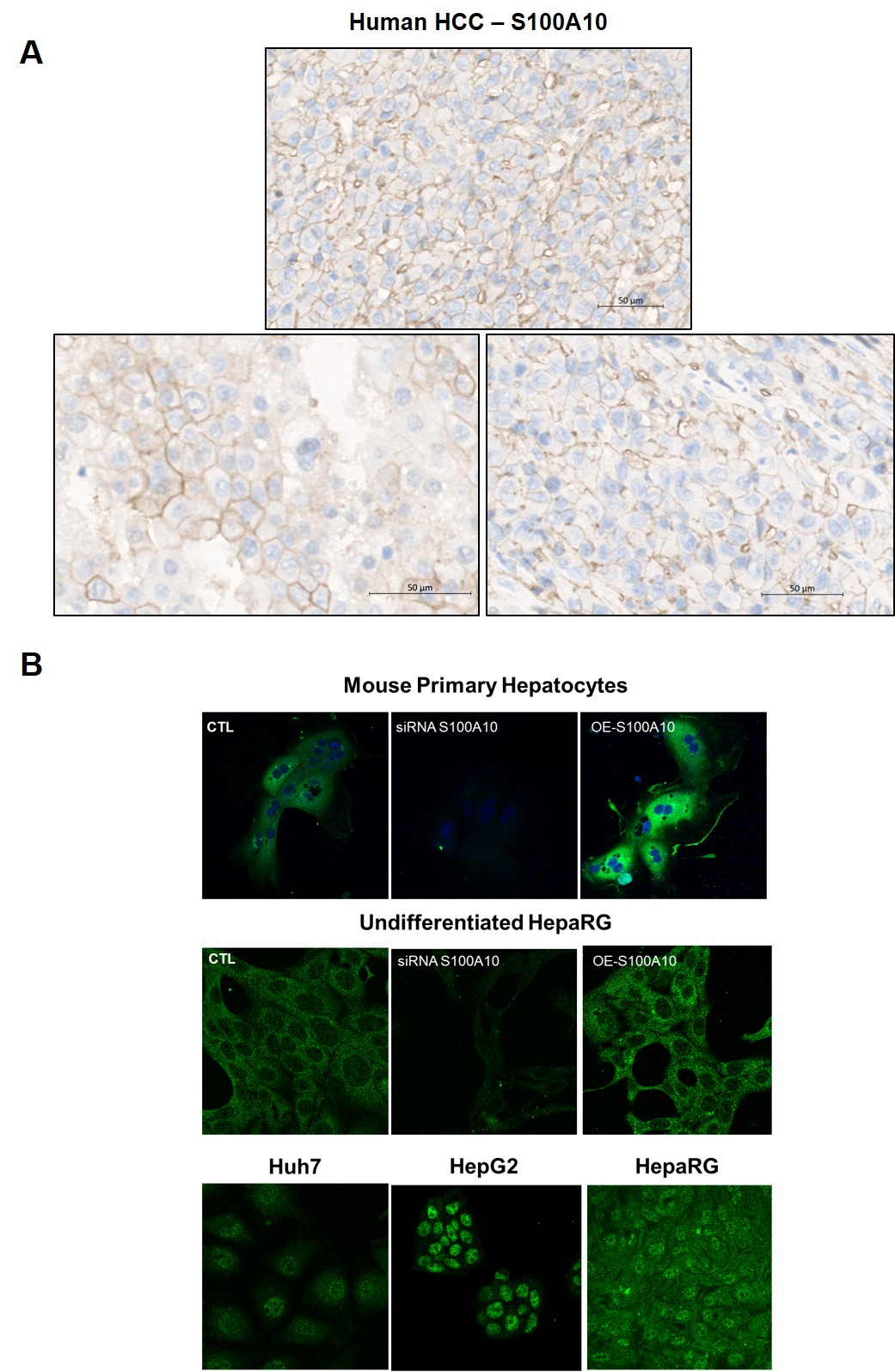

Supplement: Supplementary file 8 — Supplemental Figure 8 [file 41419_2025_7940_MOESM8_ESM.jpg]

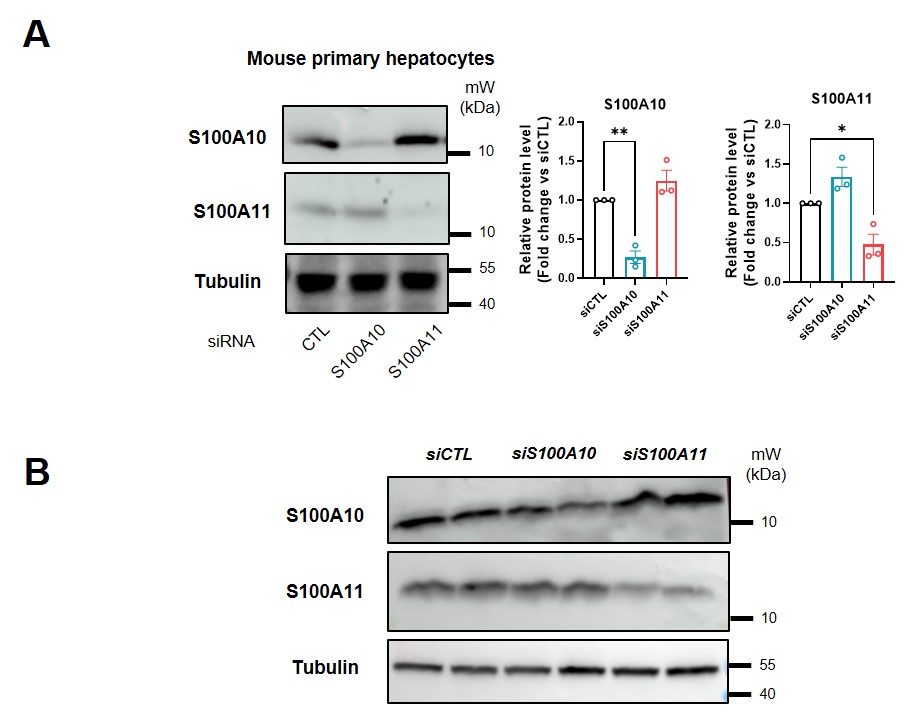

Supplement: Supplementary file 9 — Supplemental Figure 9 [file 41419_2025_7940_MOESM9_ESM.jpg]
