# Supplementary material for: Comparative analysis of S100A10 and S100A11 in MASLD and hepatic cancer development revealed a tumor suppressive role for S100A10
Source: Cell Death Dis. 2025 Aug 21;16(1):633. doi: 10.1038/s41419-025-07940-2 (PMC12370981; doi:10.1038/s41419-025-07940-2)
Supplement: Supplementary file 11 — Supplemental Material and Methods [file 41419_2025_7940_MOESM11_ESM.docx]

**Supplementary material**

**Comparative analysis of S100A10 and S100A11 in MASLD and hepatic cancer development revealed a tumor suppressive role for S100A10**

Etienne Delangre^1,#^, Marta Correia de Sousa^1^, Miranda Türkal^1^, Monika Gjorgjieva^1^, Suzanne Chartier^2^, Grégoire Arnoux^2^, Cyril Sobolewski^1^, Margot Fournier^1^, Christine Maeder^1^, Laura Rubbia-Brandt^2^, Pierre Maechler^1^, Michelangelo Foti^1^

^1^ Department of Cell Physiology and Metabolism, Faculty of Medicine, University of Geneva, CH-1211 Geneva, Switzerland.

^2^ Service de Pathologie Clinique, Hôpitaux Universitaires de Genève, Geneva, Switzerland.

# Corresponding author

Etienne Delangre, PhD

University of Geneva, Faculty of Medicine,

1, Michel Servet, CH-1211, Geneva, Switzerland

+41 223 795 421

Etienne.delangre@unige.ch

This file contain the supplementary figure legends (page 1 to 7).

**SUPPLEMENTARY FIGURE LEGENDS**

**Figure S1**

**A:** Relative mRNA level of S100A10 measured by qPCR in the liver of C57BL/6J, 3 weeks after injection with AAV8-shCTL (n=9) or AAV8-shS100A10 (n=9). Cyclophilin A is used as housekeeping gene. Results are expressed as fold change versus the group shCTL.

**B:** Relative mRNA level of S100A10 measured by qPCR in the liver of C57BL/6J, 10 weeks after injection with AAV8-shCTL (n=12) or AAV8-shS100A10 (n=11). Cyclophilin A is used as housekeeping gene. Results are expressed as fold change versus the group shCTL.

**C:** Relative mRNA level of S100A10 measured by qPCR in the liver of C57BL/6J, 10 weeks after injection with AAV8-shCTL (n=12) or AAV8-shS100A11 (n=11). Cyclophilin A is used as housekeeping gene. Results are expressed as fold change versus the group shCTL.

**D:** Quantification of macrophages density measured by Iba1 immunohistochemistry in the liver of C57BL/6J injected with AAV8-shCTL (n=7), AAV8-shS100A10 (n=6) or AAV8-shS100A11 (n=7) and fed during 24 weeks with a fructose/palmitate/cholesterol-enriched diet (FPC). Results are expressed as percentage of Iba1+ area over the total tissue area.

**E:** Liver TNFα content measured by ELISA in C57BL/6J injected with AAV8-shCTL (n=6), AAV8-shS100A10 (n=6) or AAV8-shS100A11 (n=6) and fed during 24 weeks with a fructose/palmitate/cholesterol-enriched diet (FPC).

**F:** Relative mRNA level of inflammatory mediators measured by qPCR in the liver of C57BL/6J, 10 weeks after injection with AAV8-shCTL (n=9-11), AAV8-shS100A10 (n=9-11) or AAV8-shS100A11 (n=11). Cyclophilin A is used as housekeeping gene. Results are expressed as fold change versus the group shCTL.

Results are presented as means +/- S.E.M. “n” represents the number of animals. * = p<0.05; ** = p<0.01; **** = p<0.0001 determined by t-test (Figures S1A and S1B) Mann-Whitney test (Figure S1C) or by One-Way ANOVA followed by Dunnett’s post-hoc analysis (Figures S1D, S1E and S1F).

**Figure S2**

**A:** Volcano Plot generated with deregulated proteins identified by proteomic analysis performed in explanted livers from LPTENKO shCTL versus LPTENKO shS100A10 (n=3, left panel) or from LPTENKO shCTL versus LPTENKO shS100A11 (n=3, right panel). Downregulated proteins are represented in blue and upregulated proteins in red. Fold change (FC) to control group = log2 |0.5| and –log10 (q-value)>1.

**B:** Over-representation analysis (ORA) of enriched KEGG pathways with deregulated proteins identified by proteomic analysis of explanted livers from LPTENKO-shS100A10 as compared to LPTENKO-shCTL (left) or in the liver of LPTENKO-shS100A11 as compared to LPTENKO-shCTL (right). N=3, FDR = 0.1

**C:** Venn diagram representing identified deregulated proteins common and exclusive to the shS100A10 and shS100A11 groups.

**D:** Over-representation analysis (ORA) of enriched KEGG pathways with shared deregulated proteins identified by proteomic analysis of explanted livers from LPTENKO-shS100A10 and LPTENKO-shS100A11, as compared to shCTL group. 162 proteins, FDR = 0.1

**Figure S3**

**A:** Automatic detection of collagen deposition extent based on the Masson’s coloration in the liver of 11-months old LPTENKO injected with AAV8-shCTL (n=11), AAV8-shS100A10 (n=9) or AAV8-shS100A11 (n=10). Results are expressed as the percentage of Masson’s Trichrome positive area over the total area of the tissue. Right panel: Representative images of Masson’s Trichrome staining in LPTENKO-AAV8-shCTL, LPTENKO-AAV8-shS100A10 or LPTENKO-AAV-shS100A11. Scale bar: 200μm.

**B:** Relative mRNA level of S100A10 and S100A11 measured by qPCR in the liver of LPTENKO, 5 months after injection of AAV8-shCTL (n=8), AAV8-shS100A10 (n=7) or AAV8-shS100A11 (n=8) and 2 months after re-injection. Cyclophilin A is used as housekeeping gene. Results are expressed as fold change versus the group shCTL.

Results are presented as means +/- S.E.M. “n” represents the number of animals. * = p<0.05; **** = p<0.0001 determined by t-test (Figure S3B left panel), Mann-Whitney test (Figure S3B right panel) or by Kruskal-Wallis test followed by Dunnett’s post-hoc analysis (Figure S3A).

**Figure S4**

**A:** MRI analysis of tumor number per mice in LPTENKO injected with AAV8-shCTL (n=11-14), AAV8-shS100A10 (n=9-13) or AAV8-shS100A11 (n=11-13) at 8, 9, 10 and 11 months-old.

**B:** Tumor volume measured by MRI analysis in LPTENKO injected with AAV8-shCTL (n=32-137), AAV8-shS100A10 (n=62-241) or AAV8-shS100A11 (n=29-72) at 8, 9 and 10 months-old.

**C:** Representative Western-blot (left panel) and quantification (right panel) of γH2AX and tubulin measured in the liver of LPTENKO injected with AAV8-shCTL (n=10), AAV8-shS100A10 (n=7) or AAV8-shS100A11 (n=7) at 11 months-old.

**D:** Representative images (left panel) and quantification (right panel) of γH2AX immunohistochemistry performed in the liver of LPTENKO injected with AAV8-shCTL (n=4) or AAV8-shS100A10 (n=5), at 11 months-old.

**E:** Representative images of Hemalun-Eosine stained liver histological section with foci of cellular alteration (a), adenoma (b), HCC (c), bile duct hyperplasia (d) or cholangiocarcinoma (e).

Results are presented as means +/- S.E.M. “n” represents the number animals (Figure S4A) or the number of tumors (Figure S4B). * = p<0.05; ** = p< 0.01; *** = p<0.001 determined by Kruskal-Wallis test followed by Dunnett’s post-hoc analysis (Figures S4A 8 and 9 months and S4B), by One-Way ANOVA followed by Dunnett’s post-hoc analysis (Figures S4A 10 and 11 months and S4C) or by t-test (Figure S4D).

**Figure S5**

**A:** CT-scan analysis of tumor number per mice in DEN model, injected with AAV8-shCTL (n=13), AAV8-shS100A10 (n=14) or AAV8-shS100A11 (n=13) at 8, 9, 10 and 11 months-old.

**B:** Tumor volume measured by CT-scan analysis in DEN model injected with AAV8-shCTL (n=10-116), AAV8-shS100A10 (n=13-177) or AAV8-shS100A11 (n=5-175) at 8, 9 and 10 months-old.

Results are presented as means +/- S.E.M. “n” represents the number of animals (Figure S5A) or the number of tumors (Figure S5B). * = p<0.05 determined by Kruskal-Wallis test followed by Dunnett’s post-hoc analysis (Figure S5A and S5B).

**Figure S6**

**A:** Body weight measured in DEN model, after 6 months feeding or not with 60% High Fat Diet (HFD) in the groups shCTL (n=12), shS100A10 (n=12) and shS100A11 (n=11).

**B:** Body weight gain calculated in DEN model, after 6 months feeding with 60% High Fat Diet (HFD) in the groups shCTL (n=12), shS100A10 (n=12) and shS100A11 (n=11).

**C:** MRI analysis of tumor number per mice in DEN-HFD model, injected with AAV8-shCTL (n=11-12), AAV8-shS100A10 (n=11-12) or AAV8-shS100A11 (n=11-13) at 6 and 7 months-old.

**D:** Tumor volume measured by MRI analysis in DEN-HFD model injected with AAV8-shCTL (n=39-143), AAV8-shS100A10 (n=166-421) or AAV8-shS100A11 (n=43-106) at 6 and 7 months-old.

Results are presented as means +/- S.E.M. “n” represents the number of animals (Figure S6A, S6B and S6C) or the number of tumors (Figure S6E). ** = p<0.01; **** = p<0.0001 determined by One-Way ANOVA followed by Dunnett’s post-hoc analysis (Figures S6A and S6B) or by Kruskal-Wallis test followed by Dunnett’s post-hoc analysis (Figures S6C and S6D).

**Figure S7**

**A:** Heatmap representation of proteomic analysis performed in the liver of 11 months-old LPTENKO injected with AAV8-shCTL (n=3), AAV8-shS100A10 (n=3) or AAV8-shS100A11 (n=3). Data are represented as fold change as compared to the average of shCTL condition.

**B:** Scoring of S100A10 immunohistochemistry performed on liver TMA from IHC patients comparing IHC (n=6 two per patient) and adjacent non-tumoral tissue (n=3, one per patient). Results are expressed as the percentage of low, medium or high intensity of the staining.

**C:** Scoring of S100A11 immunohistochemistry performed on liver TMA from IHC patients comparing IHC (n=6 two per patient) and adjacent non-tumoral tissue (n=3, one per patient). Results are expressed as the percentage of low, medium or high intensity of the staining.

**D:** Scoring of S100A11 immunohistochemistry performed on liver TMA from HCC patients comparing Grade 1-2 HCC (n=12 two per patient), Grade 2 HCC (n=50 two per patient), Grade 2-3 (n=6 two per patient), Grade 3 (n=24 two per patient) and adjacent non-tumoral tissue (n=42, one per patient). Results are expressed as the percentage of low, medium or high intensity of the staining.

**E:** Scoring of S100A11 immunohistochemistry performed on liver TMA from HCC patients comparing HCC from women (W, n=12 two per patient) and adjacent non-tumoral tissue (n=6, one per patient) or HCC from men (M, n=76 two per patient) and adjacent non-tumoral tissue (n=38 one per patient). Results are expressed as the percentage of low, medium or high intensity of the staining.

**F:** Scoring of S100A10 immunohistochemistry performed on liver TMA from HCC patients comparing Grade 1-2 HCC (n=12 two per patient), Grade 2 HCC (n=50 two per patient), Grade 2-3 (n=6 two per patient), Grade 3 (n=24 two per patient) and adjacent non-tumoral tissue (n=42, one per patient). Results are expressed as the percentage of low, medium or high intensity of the staining.

**G:** Scoring of S100A10 immunohistochemistry performed on liver TMA from HCC patients comparing HCC from women (W, n=12 two per patient) and adjacent non-tumoral tissue (n=6, one per patient) or HCC from men (M, n=76 two per patient) and adjacent non-tumoral tissue (n=38 one per patient). Results are expressed as the percentage of low, medium or high intensity of the staining.

**H:** Scoring of S100A10 hepatocyte localization (no staining, cytoplasm or cytoplasm/membrane) performed on liver TMA from HCC patients comparing HCC (n=92, two per patient) and adjacent non-tumoral tissue (n=46, one per patient). Results are expressed as the percentage of none, cytoplasm only or cytoplasm/membrane staining of S100A10 in hepatocytes.

Results are presented as means +/- S.E.M. “n” represents the number of animals (Figure S7A), or the number of human specimens analyzed (Figure S7B-H).

**Figure S8**

**A:** Representative images of S100A10 immunohistochemistry performed on liver TMA from 3 different HCC patients.

**B:** Immunofluorescent staining of S100A10 (green) performed in cultured Mouse Primary Hepatocytes (MPH, upper panel), in undifferentiated HepaRG (middle panel), and in Huh7, HepG2 and differentiated HepaRG (lower panel).

**Figure S9**

**A:** Representative western blot (left panel) and quantification (right panel) of S100A10 and S100A11 assessed in mouse primary hepatocytes treated for 72h with siRNAs CTL, siRNAs anti-S100A10 or siRNAs anti-S100A10 (n=3). Tubulin was used as housekeeping protein.

**B**: Representative western blot of S100A10 and S100A11 assessed in Huh7 human hepatoma cell line treated for 72 hours with siRNAs CTL, siRNAs anti-S100A10 or siRNAs anti-S100A10 (n=2). Tubulin was used as housekeeping protein.

Results are presented as means +/- S.E.M. “n” represents the number of independent experiment (Figure S9A). * = p<0.05; ** = p<0.01 determined by One-Way ANOVA followed by Dunnett’s post-hoc analysis.
